# Sustainability assessment of agricultural rainwater harvesting: Evaluation of alternative crop types and irrigation practices

**DOI:** 10.1371/journal.pone.0216452

**Published:** 2019-05-10

**Authors:** Santosh R. Ghimire, John M. Johnston

**Affiliations:** 1 Global Sustainability and Life Cycle Consultant, LLC, Athens, Georgia, United States of America; 2 U.S. Environmental Protection Agency, Office of Research and Development, Computational Exposure Division, Athens, Georgia, United States of America; University of California Davis, UNITED STATES

## Abstract

Rainwater harvesting (RWH) has been used globally to address water scarcity for various ecosystem uses, including crop irrigation requirements, and to meet the water resource needs of a growing world population. However, the costs, benefits and impacts of alternative crop types and irrigation practices is challenging to evaluate comprehensively. We present an assessment methodology to evaluate the sustainability of agricultural systems as applied to a southeastern U.S. river basin. We utilized detailed, crop-level cultivation information to calculate sustainability indicators (relative to well-water irrigation) at the basin scale (6-digit Hydrologic Unit Codes). 40 design configurations comprising crop types and irrigation practices were evaluated to demonstrate the methodology’s robustness. Four RWH designs and four major crops (pasture-grass, soybeans, corn, and cotton) resembling current practices were evaluated, as well as six combined systems (combined RWH and well-water systems) with four globally representative crops (corn, soybeans, wheat, and quinoa). Sustainability scores were calculated by integrating seven life cycle impact indicators (cumulative energy demand, CO_2_ emission, blue water use, ecotoxicity, eutrophication, human health-cancer, and life cycle costs). At a basin-wide RWH adoption rate of 25%, the benefits, relative to 100% well-water, of the RWH systems irrigating soybeans and supported with well-water (0.4 well-water: 0.6 RWH) provided cumulative energy savings of 39 Peta Joule and reductions in CO_2_ emission, blue water use, ecotoxicity, eutrophication, and human health-cancer at 1.9 Mt CO_2_ eq., 6.9 Gm^3^, 5.7 MCTU, 6.6 kt N eq., and 0.07 CTU, respectively. These benefits increased linearly with RWH scaling variables including the adoption rates, system service life, crop area, and water needs. Our methodology integrates the three pillars of agricultural sustainability specific to rainwater harvesting into a single score. It is applicable to other locations worldwide facing water scarcity by modifying the RWH system design, selecting other crop types, and obtaining appropriate data.

## Introduction

Humanity faces many challenges related to drinking water, energy, and agricultural production due to the competing needs of water resources and growing world population. These include megadroughts, floods, and unprecedented water scarcity, as well as environmental and human health implications in the U.S. and around the world [1–5]. In addition, increasing anthropogenic activities such as urbanization, industrialization, and intensification of agriculture lead to water scarcity for households, and other sectors [2, 6–10]. Globally, “nearly 80% of the world’s population is exposed to high levels of threat due to water security” [6]. The southeastern U.S., like many areas, has been impacted by droughts and floods, as evidenced by heavy downpours in the autumn and moderate-to-severe droughts in spring and summer seasons (12% and 14%, respectively), between 1970 and 2007 [11].

Human health depends on ecosystem health, e.g., careful management of water resources is important to sustain the health of society and agricultural sustainability [12]. Water supply for agricultural irrigation can be a huge challenge due to the global agricultural freshwater withdrawal of 70%, excessive groundwater abstraction, and a doubling in food demand by 2050 [13–17], in addition to the climate change effects on agricultural production [18, 19]. Coping with these challenges requires sustainable agricultural systems adapting alternative food crops and irrigation practices. Alternative food crops such as buckwheat, barley, and quinoa are highly nutritious with dry weight crude protein of 18.5% (buckwheat), 14.7% (barley) and 13.8% (quinoa), compared to corn (8.7%) and wheat (13%) [20]. Alternative irrigation practices involve replacing or augmenting conventional sources such as well water with decentralized “soft-path” [21] alternatives, e.g., rainwater. The use of rainwater is recognized as a viable alternative, supplementing conventional supplies to meet demands for drinking, washing, sanitation, and crop irrigation, in addition to alleviating potential droughts in the face of climate change [22–27]. This is in addition to environmental co-benefits such as mitigated sewer overflows, increased food and economic security, and reduced human and environmental impact [28–30]. A number of studies have addressed various aspects of RWH including the design, economic assessment and cost efficiency, water efficiency, life cycle impacts assessments, and eco-efficiency evaluation [31–37]. Others conducted hydrologic impact modeling and reported RWH as potential decentralized water storage option for supplemental irrigation and climate change adaptation [38–40]. However, the RWH design, performance, and associated cost and environmental impacts vary with regions, climate type, and annual rainfall trends in addition to rainwater uses [30, 41]. Moreover, the costs, benefits and impacts of agricultural systems are challenging to evaluate comprehensively. Scientific questions at this initial stage are: Are agricultural systems sustainable at the basin scale (6-digit Hydrologic Unit Codes, HUCs), and are certain crops types and irrigation practices more, or less suitable? Can we provide RWH practitioners and policy makers with sustainability indicators to inform decision making? And most importantly, can we provide a generalizable methodology for the characterization of agricultural sustainability indicators?

Sustainability can be defined in many ways, but the core definition consists of economic, environmental, and social indicators of system/product/service that dictate needs of both present and future generations [42–47]. Sustainability science deals “with the interactions between natural and social systems” [48–51] by incorporating multidisciplinary indicators [47]. In the context of agricultural sustainability, the National Research Council (NRC) and the Food, Agriculture, Conservation, and Trade Act (FACTA) of 1990 highlighted environmental quality, natural resources, efficient use of nonrenewable resources, quality of life, and economic viability as indicators [52, 53]. Ghimire and Johnston [47] presented a sustainability assessment methodology and modified eco-efficiency framework comprised of four economic, 11 environmental, and three social indicators for use in water resource management. Relevant previous work and the refinement to current study are summarized in Table 1 [30, 47, 54, 55].

**Table 1:** Summary of previous studies and refinement to current study.

Previous study	Synthesis	Refinement for current study
Hydrologic assessment of RWH at the watershed-scale [54]	Conducted hydrologic impacts assessment of domestic and agricultural RWH systems for corn crop irrigation at adoption rates of 25, 50, 75, and 100% within the Albemarle-Pamlico (A-P) basin (12-digit Hydrologic Unit Code-level). Reported that a 25% adoption rate reduced downstream average monthly water yield by as little as 6%.	The design of agricultural RWH systems was adopted to the scale of A-P basin. The supplemental irrigation water needs for corn crop were used as a reference to estimate water demands for all crops in the region.
Life cycle assessment (LCA) of rainwater harvesting (RWH) [30]	Performed LCA of RWH systems, and compared the life cycle impact assessment (LCIA) categories of functional unit rainwater supply (impacts/m^3^) to conventional water supplies, municipal drinking water and well-water irrigation. Reported that two minimal designs with no pumps reduced environmental impact, from 78% energy use to 88% human health criteria pollutants.	LCIAs of four configurations of agricultural RWH systems were adopted to complete the LCIAs of 16 decision management objectives (DMOs) at the A-P basin level.
Holistic impacts assessment of RWH at the watershed scale [55]	Presented holistic impacts (economic and environmental) of domestic and agricultural RWH systems by scaling up functional unit LCIA impacts at adoption rates of 25, 50, 75, and 100% in three diverse watersheds within the A-P basin.	The holistic approach was modified to evaluate sustainability of agricultural RWH at the A-P basin scale at a 25% adoption rate.
Eco-efficiency framework and sustainability analysis methodology for green infrastructure practices [47]	Presented a modified eco-efficiency framework and demonstrated sustainability methodology to analyze 20 domestic RWH designs. Also addressed subjectivity and sensitivity analysis requirements of sustainability analysis, and evaluated performance of 10 weighting schemes that included classical data envelopment analysis (DEA), equal weights, National Institute of Standards and Technology’s stakeholder panel, Eco-Indicator 99, Sustainable Society Foundation’s Sustainable Society Index, and five derived threshold schemes.	The framework was used to select sustainability indicators, and the equal weights scheme was employed in sustainability assessment of agricultural systems. We advanced the methodology by calculating comparative sustainability indicators of agricultural systems (summarized later).

### 1.1 Objectives, scope, and novelty

The current study aims to develop a general methodology for sustainability assessment of agricultural systems (various design configurations of crop types and irrigation practices). The methodology is demonstrated in the Albemarle-Pamlico river basin (6-digit HUCs) located in the southeastern U.S.

Two groups of 40 alternative design configurations are defined as decision management objectives (DMOs) consistent with Ghimire and Johnston [47], comprising crop types and irrigation practices. Group 1’s 16 DMOs represent four RWH design configurations and the basin’s major crops. Group 2’s 24 DMOs represent six combined systems (RWH and well-water) and four globally representative crops. The basin’s major crops are pasture-grass, soybeans, corn, and cotton. The globally representative crops are corn, soybeans, wheat, and quinoa [56], which are relevant to food-insecure regions of Asia, Africa, and South and Central America [5]. Among globally representative crops, quinoa is not cultivated in the southeastern U.S. However, it was selected in anticipation of wider use in the U.S. Importantly, quinoa is recognized not only for its value in reducing dependence on staples like wheat and rice [57] but also drought tolerance. Quinoa has an average annual water requirement of 317.5 mm per year [20], about half that of corn, and is already produced in Colorado and Nevada [58].

Using the modified eco-efficiency framework [47], seven sustainability indicators were defined to compare the DMOs with well-water irrigation. Life cycle impact assessment (LCIA) category values and life cycle cost assessment (LCCA) values per functional unit of 1 m^3^ water delivery by a system were used as indicators. Using a standard unit facilitated comparison of a DMO to well-water irrigation system. The indicators included cumulative energy demand, CO_2_ emission, blue water use, ecotoxicity, eutrophication, life cycle cost, and human health-cancer impact. Holistic sustainability scores were calculated using modified data envelopment analysis (DEA), a widely used statistical method, with equal weights to environmental/human health indicators [47]. Functional unit sustainability indicators for each DMO were scaled basin wide.

While a number of studies have addressed design, economic impacts, water efficiency, life cycle environmental impacts, and eco-efficiency of RWH, our methodology, for the first time, integrates the three pillars of agricultural systems sustainability specific to rainwater harvesting into a single score at the basin scale. The following sections describe methods, tools, and results and discussion with a summary on study implications. Additional details on agricultural systems as DMOs, LCIA, LCA, and DEA are provided in the S1 Supporting Information.

## Methodology and tools

**Fig 1:**
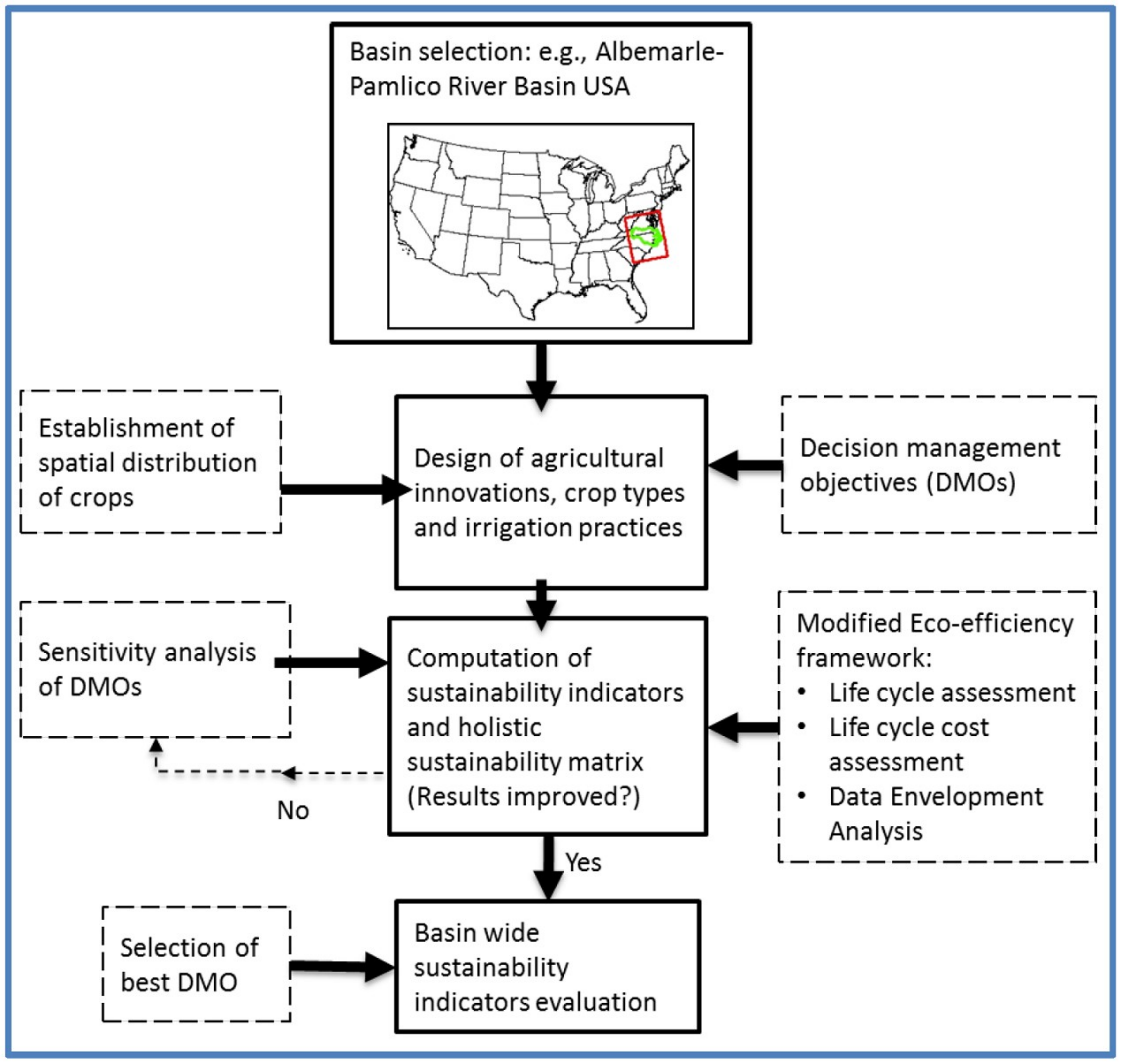
Workflow of sustainability assessment of basin-wide agricultural systems in the Albemarle-Pamlico. The middle three components (solid boxes) are supported with corresponding dotted boxes, culminating in a basin-wide sustainability indicator evaluation.

The general procedure of sustainability assessment is depicted in a flow diagram (Fig 1) and illustrated below.

### 2.1 Basin selection

**Fig 2:**
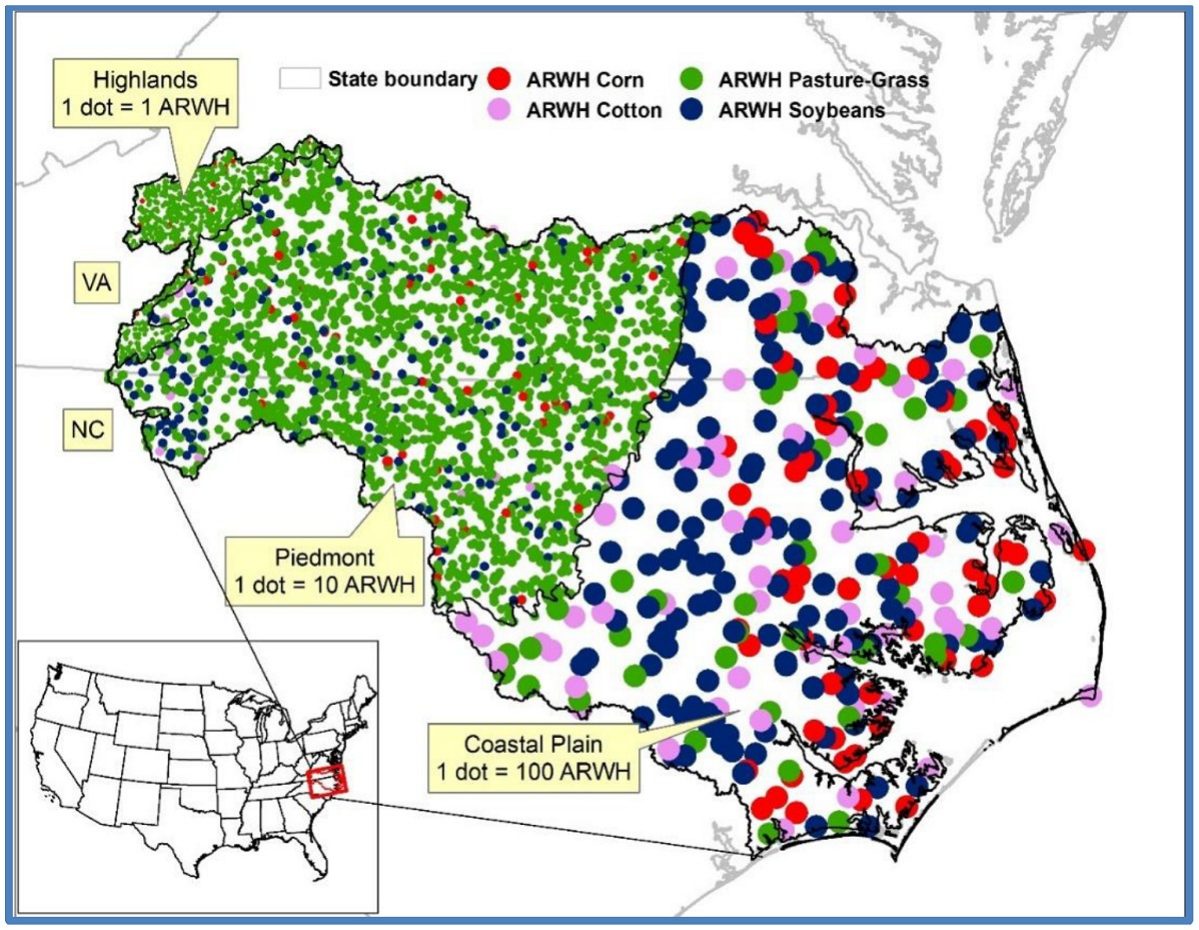
Agricultural RWH system for irrigation to major crops in areas greater than 10% of total farm within the Albemarle-Pamlico basin (Figure modified from Ghimire and Johnston [55]; ARWH = agricultural RWH).

**Fig 3:**
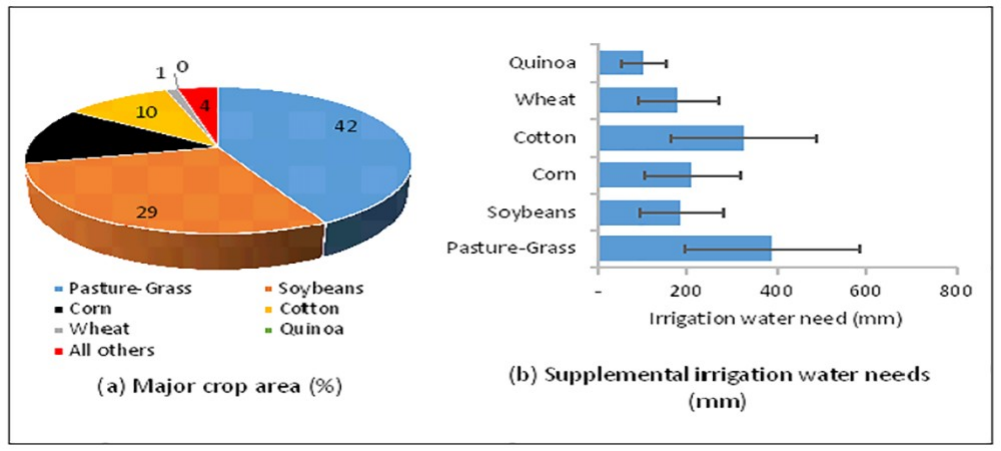
(a) Crop areas and (b) estimated annual supplemental irrigation water needs for the crops (>10% area coverage) with ±50% variation in demands in this study. “All others” include but are not limited to tobacco, peanuts, sorghum, potatoes, millet, oats, rye, barley, triticale, and fruits and vegetables such as cucumbers, watermelons, peppers, etc. Note: Quinoa is currently not cultivated in the region; however, we assumed the area equivalent to wheat’s area.

The Albemarle-Pamlico river basin located in North Carolina (NC) and Virginia (VA) was selected because it has been well-studied [30, 59] and has a wealth of data including watershed characteristics and crops (Figs 2 and 3). The basin extends from VA (80°27'16.967" W 37°4'3.62" N) to NC (75°42'56.239" W 35°38'57.165" N).

Annual crop water need (including water necessary for transpiration and evaporation, or evapotranspiration, ET) is defined as the sum of supplemental water need (irrigation) and the net available or effective rainwater for the crop to grow optimally [60]. Effective rainwater (net rainwater available to a crop) is total precipitation, minus percolation below the plant root zone, minus runoff over the soil surface. Note that crop water need depends on temperature, humidity, wind speed, cloud cover, rainfall amount, crop type, and crop growth stage. Various methods—experimental (using an evaporation pan) or theoretical (using measured climatic data such as the Blaney-Criddle method)—are available to estimate evapotranspiration [60]. For simplicity, we used the Food and Agriculture Organization (FAO) of the United Nations [60] approximated values of seasonal crop water needs to estimate irrigational crop water needs.

### 2.2 Design of basin-wide agricultural systems as decision management objectives (DMOs)

The Cropland Data Layer (CDL) of the U.S. Department of Agriculture (USDA) [61] was used to establish a spatial distribution of crop area at resolution of 30 m x 30 m and to identify major crops within the basin (>10% area coverage, Fig 2). For simplicity and to avoid double-counting, only the follow-up crop was considered in double-cropping (Dbl Crop), i.e., harvest of two crops from the same field in a given year. For example, “Dbl Crop WinWht/Soybeans” means double-cropping with Winter Wheat, followed by Soybeans [62]; “Dbl Crop WinWht/Soybeans” and “Dbl Crop Barley/Soybeans” were counted as Soybeans; “Dbl Crop Barley/Corn”, “Dbl Crop WinWht/Corn”, “Dbl Crop Oats/Corn”, and “Sweet Corn” as Corn; “Dbl Crop WinWht/Cotton” and “Dbl Crop Soybeans/Cotton” as Cotton. Also “Grass/Pasture”, “Sod grass seed”, “Hay” (alfalfa and non-alfalfa), and “Switchgrass” were considered as Pasture-Grass.

Two Groups of 40 agricultural systems (comprising alternative crop types and irrigation practices of agricultural RWH combined with well-water irrigation) as DMOs were developed to illustrate the methodology. Group 1’s 16 DMOs were derived from four RWH design configurations for irrigating the basin’s four major crops (pasture-grass, soybeans, corn, and cotton) (Table 2).

**Table 2:** Description of Cluster 1 decision management objectives (DMOs). Note: baseline system consisted of RWH components: 13000 m^3^ sediment chamber, 155 m 101.6 mm diameter collection and distribution polyvinyl chloride (PVC) pipe, a polyethylene (PE) water holding tank, a pump, pumping energy, a pivot-center, control valves, and check valves, designed for corn crop irrigation adopted from Ghimire et al. (2014). All DMOs were a modification of the baseline system.

Description of Cluster 1 DMOs	Notation
Baseline System Pasture-Grass irrigation	DMO1
Baseline System Cotton irrigation	DMO2
Baseline System Corn (Reference crop) irrigation	DMO3
Baseline System Soybean irrigation	DMO4
Concrete Tank System Pasture-Grass irrigation	DMO5
Concrete Tank System Cotton irrigation	DMO6
Concrete Tank System Corn irrigation	DMO7
Concrete Tank System Soybean irrigation	DMO8
No pump System PE Tank Pasture-Grass irrigation	DMO9
No pump System PE Tank Cotton irrigation	DMO10
No pump System PE Tank Corn irrigation	DMO11
No pump System PE Tank Soybean irrigation	DMO12
No pump System Concrete Tank Pasture-Grass irrigation	DMO13
No pump System Concrete Tank Cotton irrigation	DMO14
No pump System Concrete Tank Corn irrigation	DMO15
No pump System with concrete Tank Soybean irrigation	DMO16

A previously designed agricultural RWH system for corn crop irrigation [30] (hereafter, baseline system) was modified for four RWH configurations: Configuration 1: baseline system; Configuration 2: Concrete tank system; Configuration 3: no pump PE tank system; and Configuration 4: no pump concrete tank system. The four major crops (>10% crop area) were pasture-grass, cotton, corn, and soybeans, with corresponding average water needs at 1,200 mm/y, 1,000 mm/y, 650 mm/y, and 575 mm/y [60]. The baseline system included 13000 m^3^ sediment chamber, 155 m long 101.6 mm diameter collection and distribution polyvinyl chloride (PVC) pipe, a polyethylene (PE) water-holding tank, a pump, pumping energy at 0.30 kWh/m^3^, a pivot-center, control valves, and check valves; all other configurations were obtained by modifying, replacing or eliminating the baseline system components; for example, Configuration 2’s Concrete tank system replaced the PE tank of the baseline system.

The methodology was extended to evaluate sustainability of alternative, representative food crops in the southeastern U.S. and globally-prioritized crops in food-insecure regions. These included corn, soybeans, wheat and quinoa. Group 2’s 24 DMOs were derived from four crops and six optimal RWH systems combined with well water; the six combined systems (combined RWH and well-water systems) were supported with a fraction of well water at 0.00, 0.20, 0.40, 0.60, 0.80, and 1.00 (Table 3). An optimal agricultural RWH system was created by eliminating the water holding tank and pump.

**Table 3:** Description of Cluster 2 decision management objectives (DMOs).

Description of Cluster 2 DMOs	Notation
0%RWH-Corn	DMO1
0%RWH-Soybeans	DMO2
0%RWH-Wheat	DMO3
0%RWH-Quinoa	DMO4
20%RWH-Corn	DMO5
20%RWH-Soybeans	DMO6
20%RWH-Wheat	DMO7
20%RWH-Quinoa	DMO8
40%RWH-Corn	DMO9
40%RWH-Soybeans	DMO10
40%RWH-Wheat	DMO11
40%RWH-Quinoa	DMO12
60%RWH-Corn	DMO13
60%RWH-Soybeans	DMO14
60%RWH-Wheat	DMO15
60%RWH-Quinoa	DMO16
80%RWH-Corn	DMO17
80%RWH-Soybeans	DMO18
80%RWH-Wheat	DMO19
80%RWH-Quinoa	DMO20
100%RWH-Corn	DMO21
100%RWH-Soybeans	DMO22
100%RWH-Wheat	DMO23
100%RWH-Quinoa	DMO24

### 2.3 Evaluation of sustainability indicators and holistic sustainability

For the purpose of current study, a holistic sustainability indicator was defined by integrating economic indicator with environmental and social indicators, consistent with Ghimire and Johnston [47]. Life cycle impact assessment (LCIA) category values and life cycle cost assessment (LCCA) values per functional unit of 1 m^3^ water delivery were selected as sustainability indicators, and the two Groups’ DMOs were evaluated consistent with the modified eco-efficiency framework [47]. The LCIA values included cumulative energy demand, CO_2_ emission, blue water use, ecotoxicity, eutrophication, human health-cancer, and the LCCA value was life cycle costs. More specifically, the life cycle reduction in blue water use, cumulative energy savings, reduction in CO_2_ emission, in addition to reduced human health impact, ecotoxicity, and eutrophication embraced the definition of agricultural sustainability used by NRC and FACTA towards sustainable agricultural systems [52, 53].

#### 2.3.1 LCIA and LCCA

A prior LCA study [30] provided the LCIA categories per functional unit of 1 m^3^ water delivery (impacts/m^3^) of the four RWH configurations, the optimal RWH system, and well-water irrigation system for the reference crop (corn). The OpenLCA tool was utilized for the LCA calculations in conjunction with the USEPA’s LCIA methods, Tool for the Reduction and Assessment of Chemical and Other Environmental Impacts (TRACI 2.0), and ReCiPe method [30, 63–65]. Necessary life cycle inventory data was compiled from the Building for Environmental and Economic Sustainability (BEES) [66] and the ecoinvent database version 2.2 [67].

LCCA per m^3^ water delivery ($/m^3^) of these agricultural RWH and well-water irrigation systems were performed as the sum of the present values of investment costs, energy costs, operation and maintenance costs, and residual values over the lifetime consistent with Ghimire and Johnston [47] and Fuller and Petersen [68]. Description of LCCA is provided in S1 Supplementary Information.

LCIA and LCCA values of 16 DMOs of Group 1 (L_C1_) were estimated as a function of ∝-parameter (the ratio of crop water need to reference crop water need):

$${\text{L}}_{\text{C}1}=\propto \;\text{x}\;L_{\text{config}.}$$ (1)

where

$$\propto =\;\frac{S_{c}}{S_{rc}}=\frac{W_{c}}{W_{rc}}$$ (2)

S_c_ = seasonal (i.e., annual) supplemental water need for a crop, m

S_rc_ = seasonal (annual) supplemental water need for the reference crop (corn), obtained from Ghimire and Johnston [54], m

W_c_ = seasonal (annual) water need for an actual crop, m

W_rc_ = seasonal (annual) water need for the reference crop, m

*W*_*c*_ and *W*_*rc*_ obtained from the Food and Agriculture Organization’s Irrigation Water Management Training Manual [60], except for Quinoa [20] which is not currently cultivated in the Albemarle-Pamlico Basin (Table 4)

L_config._ = the LCIA and LCCA values of a RWH Configuration

Similarly, the LCIA (impacts/m^3^) and LCCA values of Group 2 DMOs (L_C2_) were estimated as:

$${\text{L}}_{\text{C}2}=\;\propto {\;\text{x}\;\text{L}}_{\text{com}}$$ (3)

where

L_com_ = the LCIA and LCCA values of combined system of RWH and well-water, and

$$L_{com}=\;p_{a}\;\text{x}\;L_{a}+p_{w}\;\text{x}\;L_{w}$$ (4)

where

*L*_*a*_ = the optimal agricultural RWH system’s life cycle impact (impact/m^3^), or life cycle costs ($/m^3^)

*L*_*w*_ = the well-water system’s life cycle impact (impact/m^3^), or life cycle costs ($/m^3^)

*p*_*a*_ and *p*_*w*_ are optimal agricultural RWH and well-water percentage (in decimal) such that

$$p_{a}+p_{w}=1$$ (5)

**Table 4:** A summary of cropland, supplemental water needs, and RWH systems in the research site. *S*_*c*_ = *αS*_*rc*_ where, *α* = the ratio of crop water need to reference crop water need, *S*_*c*_ = supplemental irrigation water need; *S*_*rc*_ = the supplemental irrigation water need for the reference crop, corn (211.3 mm), obtained from Ghimire and Johnston [54]: HIGH = Highlands; PIED = Piedmont; COAS = Coastal; N/A = not available. Note: the number of agricultural RWH systems irrigating a specified crop, *i*, was calculated as the ratio of actual total crop area to average unit farm area (343,983 m^2^, obtained from Ghimire and Johnston [54]. Although quinoa is currently not cultivated in the region, analyses were conducted equivalent to wheat’s area.

Cropland cover type	α- parameter	Supplemental water need, *S*_*c*_	Total crop area in each physiographic province				Number of agricultural RWH systems in each physiographic province		
Crop, *i*		(m/y)	HIGH (km^2^)	PIED (km^2^)	COAS (km^2^)	Total (km^2^)	HIGH	PIED	COAS
Pasture-Grass	1.85	0.39	274.4	6,055.7	1,732.9	8,063.0	798	17,605	5,038
Cotton	1.54	0.325	0.0	40.6	1,962.1	2,002.7	-	118	5,704
Corn	1	0.211	5.4	203.6	2,228.9	2,437.9	16	592	6,480
Soybeans	0.88	0.187	0.3	715.6	4,856.5	5,572.4	1	2,080	14,119
Wheat	0.85	0.179	0.4	113.0	113.3	226.7	1	328	329
Quinoa	0.49	0.103	N/A	N/A	N/A	N/A	N/A	N/A	N/A

In Eq (4), the L_com_ values were calculated using the functional unit impacts of both optimal RWH and well-water irrigation systems for the reference crop, by summing the fractional values of both systems delivering 1 m^3^ of water supply consistently with a previously published approach [32]. Here, we note that the LCIA and LCCA values per functional unit of 1 m^3^ water delivery were calculated using normalized flow inputs with respect to volumetric water supply, and that input amounts were linearly related to volumetric water supply.

For consistency, LCIA values of well-water irrigation systems, comparable to all DMOs of each Group, were also estimated as function of ∝-parameter by multiplying LCIA values of well-water system for the reference crop irrigation by ∝.

#### 2.3.2 Data envelopment analysis

The functional unit LCIA and LCCA values of the DMOs of each Group were mean normalized and holistic sustainability scores were calculated by integrating these indicators using modified Data Envelopment Analysis (DEA), consistent with Ghimire and Johnston [47]. The classical DEA formulation began with standard eco-efficiency as the economic output divided by the linear function of environmental input (Eq 6) [47, 69].

$$Maximize\;\;E_{n}=\frac{A_{n}}{w_{1}D_{n1}+w_{2}D_{n2}+\ldots .+w_{X}D_{nX}}\;\text{(for}\;\text{all}\;\text{n}\;=\;1\;\text{to}\;\text{N)}$$ (6)

subject to

$$\frac{A_{1}}{w_{1}D_{11}+w_{2}D_{12}+\ldots .+w_{X}D_{1X}}\leq 1$$ (7)

$$\frac{A_{2}}{w_{1}D_{21}+w_{2}D_{22}+\ldots .+w_{X}D_{2X}}\leq 1$$ (8)

$$\frac{A_{N}}{w_{1}D_{N1}+w_{2}D_{N2}+\ldots .+w_{X}D_{NX}}\leq 1$$ (9)

$$w_{1},\;w_{2},\ldots w_{X}\geq 0$$ (10)

where,

*E* = holistic sustainability score

*A* = economic indicator

*D* = environmental indicator

*w*_*i*_ = model weight estimated by DEA optimization, *i* ranges from 1 to *X*, the number of environmental and social impacts (in this example, *X* = 6).

And, the *n*th DMO of *N* DMOs induced *X* environmental impacts, measured by D_*nX*_. Each DMO had one economic indicator, *A*_*n*_.

These non-linear equations were transformed to linear form by determining the inverse functions, the classical DEA was improved by incorporating the equal weighting scheme for each DMO, and holistic sustainability scores were estimated. The most sustainable DMO pertaining to each crop type was identified to evaluate optimal LCIA benefits of basin-wide agricultural RWH sustainability. Detail description of DEA formulation and solution is provided in S1 Supporting Information.

### 2.4 Estimation of sustainability indicators of basin-wide agricultural systems

The functional unit sustainability indicators of the agricultural systems (i.e., DMOs comprising alternative crop types and irrigation practices of agricultural RWH combined with well-water irrigation) were scaled to basin-wide indicators, using Eq (11).

$$I_{j}=T\;\text{x}\;R_{j}\;\text{x}\;S_{j}\;\;\Delta i_{j}\;\text{x}\;\sum_{p}A_{jp}$$ (11)

where

*I*_*j*_ = change or net benefits in the basin-wide impacts of a DMO with respect to conventional well-water irrigation impacts for crop *j* (Units: Energy demand (MJ); CO_2_ emission (kg CO_2_ eq); Blue water use (m^3^); Ecotoxicity (CTU or comparative toxic units); Eutrophication (kg N eq); and Human health-cancer (CTU)

*T =* service life of a DMO (50 y)

*R*_*j*_ = agricultural RWH system adoption rate basin wide (in decimal, 0.25)

*S*_*j*_ = seasonal (annual) supplemental water need for crop *j*, (m/y) (Eq 2)

*Δi*_*j*_ = change in LCIA values of DMOs with respect to conventional irrigation impacts (Units/m^3^) for crop *j* irrigation, adopted from prior LCA study [30]

*A*_*jp*_ *=* specified crop area (m^2^) in each physiographic province (denoted by the suffix *p)* estimated based on CDL database. There are three provinces within the basin: Highlands (H), Piedmont (P), and Coastal Plain (C).

For a specific agricultural RWH crop irrigation, Eq 11 reduces to:

$$I_{j}=T\;\text{x}\;R_{j}\;\text{x}\;S_{j}\;\;\Delta i_{j}\text{x}\;\left(A_{jH}+A_{jP}+A_{jC}\right)$$ (12)

where,

*A*_*jH*_ *+ A*_*jP*_ *+ A*_*jC*_ = sum of crop *j* area (m^2^) in the provinces

It is noted that quinoa was not reported in the Albemarle-Pamlico basin; however, we discussed basin-wide sustainability indicators for quinoa irrigation by considering a hypothetical crop area equivalent to wheat.

Sustainability indicators of most sustainable DMOs for each crop type at a reasonable adoption rate of 25% basin wide were described. Sustainability indicators of a hypothetical combined system (RWH combined with well water at a fraction of 0.4) were also discussed. Sensitivity of the indicators to each of the variables (Eq 12): annual supplemental water need (±50%), adoption rates (25%, 50%, 75%, and 100%), system service life (25, 50, 75, and 100 years), and crop area (±50%), was discussed, keeping remaining variables constant.

## Results and discussion

### 3.1 Basin-wide agricultural systems

The spatial distribution of crop types in the Albemarle-Pamlico river basin (Fig 2) showed pasture-grass (42%), soybeans (29%), corn (13%), and cotton (11%) were the four major crops (>10% crop area) found in the basin (Fig 2, Table 4). Estimated supplemental water needs for these major crops ranged from 0.40 m/y (pasture-grass) to 0.20 m/y (soybeans). The ratio of actual crop to reference crop water need (defined as ∝-parameter) ranged from 0.49 (quinoa) to 1.85 (pasture grass). Wheat, sorghum, peanuts, and potatoes were other crops cultivated in the region (<2% crop area); however, protein-rich crops now growing commercially in the U.S. (e.g., quinoa) were not reported in the region.

Sixteen agricultural systems as DMOs of Group 1 differed by RWH design configuration (polyethylene (PE) versus concrete tank, with/without pump) and current crop practices (pasture-grass, soybeans, corn, and cotton) in the region. Twenty-four DMOs of Group 2 differed by percentage of RWH and well-water systems (i.e., combined RWH and well-water systems) irrigating four globally representative crops (corn, soybeans, wheat, and quinoa).

### 3.2 Sustainability indicators and holistic sustainability of agricultural systems

**Fig 4:**
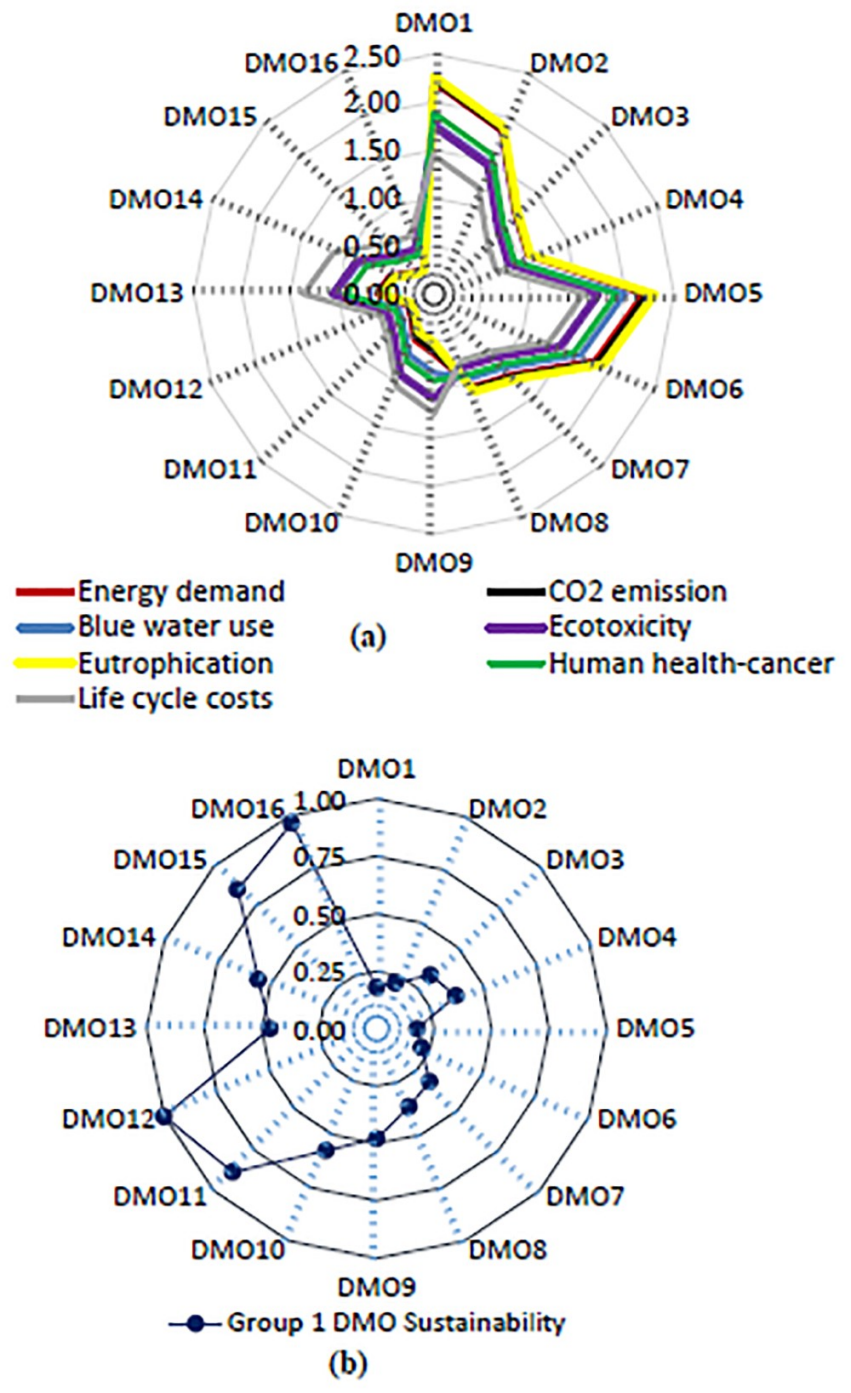
(a) Group 1- mean normalized life cycle impact assessment and life cycle cost assessment values (dimensionless) of 16 decision management objectives (DMOs) for agricultural RWH systems irrigating four major crops resembling current practices in the southeastern U.S. (b) Group 1- Sustainability scores of 16 DMOs. Note: DMO1 = baseline system pasture-grass irrigation; DMO2 = baseline system cotton irrigation; DMO3 = baseline system corn irrigation; DMO4 = baseline system soybeans irrigation; DMO5 = concrete tank system pasture-grass irrigation; DMO6 = concrete tank system cotton irrigation; DMO7 = concrete tank system corn irrigation; DMO8 = concrete tank system soybeans irrigation; DMO9 = no pump system PE tank pasture-grass irrigation; DMO10 = no pump system PE tank cotton irrigation; DMO11 = no pump system PE tank corn irrigation; DMO12 = no pump system PE tank soybeans irrigation; DMO13 = no pump system concrete tank pasture-grass irrigation; DMO14 = no pump system concrete tank cotton irrigation; DMO15 = no pump system concrete tank corn irrigation; and DMO16 = no pump system with concrete tank soybeans irrigation.

For Group 1 DMOs, the functional unit LCIA values (i.e., life cycle cumulative energy demand, CO_2_ emission, blue water use, ecotoxicity, eutrophication, human health-cancer) varied with DMO, crop type, and RWH irrigation system design (Fig 4a and S1 Supporting Information).

More specifically, DMOs with no pump (DMOs 9–16) had lower LCIA values than DMOs with pump (DMOs 1–8). Mean-normalized values of LCIA were lowest for DMO 12 (no pump system with PE tank soybean irrigation). These values were highest for systems irrigating pasture-grass. For pasture-grass irrigation, the mean-normalized life cycle energy demand ranged from 0.60 (DMO 13, no pump system with concrete tank) to 2.21 (DMO 1, baseline system), and different patterns were observed for other indicators. The mean-normalized energy demand was the lowest (0.29 for DMO 16 to 1.06 for DMO 4) for soybeans irrigation, primarily due to the lower ∝-parameter (the ratio of actual to reference crop water need) of 0.88 and 1.85 for soybeans and pasture-grass irrigations, respectively. Ranking of holistic sustainability scores showed DMO 12, 16, 11, and 15 were the top four in sustainability, with scores of 1.00, 0.97, 0.88, 0.86, respectively (Fig 4b). For the four major crops in the region, DMOs 12, 11, 10, and 9 (i.e., no pump systems with PE tank for soybeans, corn, cotton, and pasture-grass irrigation) were the most sustainable with scores of 1.00, 0.88, 0.58, and 0.48, respectively. It is noted that the ranking of the DMOs is limited by the number of DMOs and their configuration that may be different for different geographic location, rainfall/runoff potential, and system operation and maintenance requirements.

**Fig 5:**
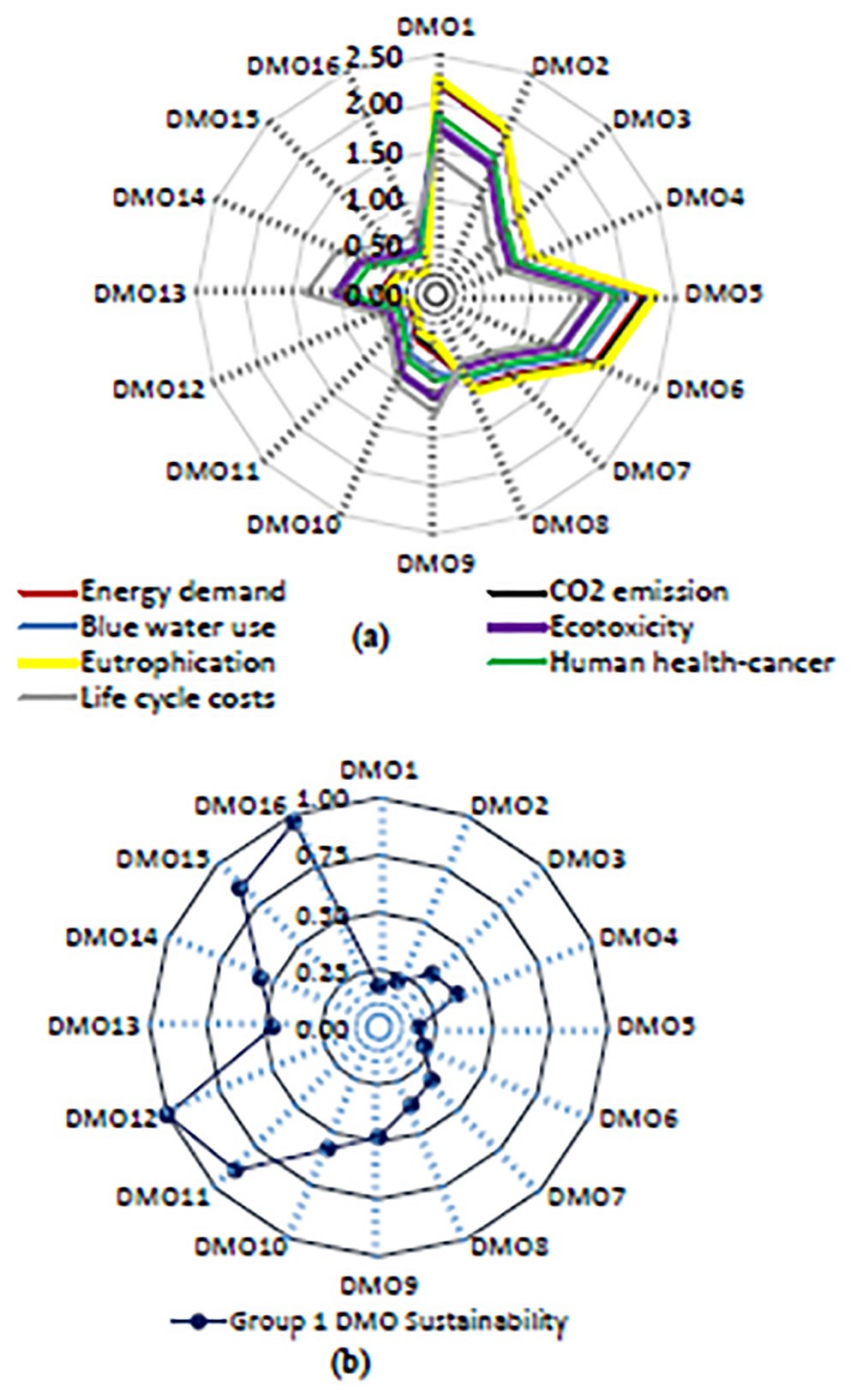
(a) Group 2- mean normalized life cycle impact assessment and life cycle cost assessment values (dimensionless) of 24 decision management objectives (DMOs) for combined systems of optimal agricultural rainwater harvesting (RWH) irrigating four representative crops (b) Group 2- Sustainability scores of 24 decision management objectives (DMOs). Note: DMO1 = 0%RWH-Corn; DMO2 = 0%RWH-Soybeans; DMO3 = 0%RWH-Wheat; DMO4 = 0%RWH-Quinoa; DMO5 = 20%RWH-Corn; DMO6 = 20%RWH-Soybeans; DMO7 = 20%RWH-Wheat; DMO8 = 20%RWH-Quinoa; DMO9 = 40%RWH-Corn; DMO10 = 40%RWH-Soybeans; DMO11 = 40%RWH-Wheat; DMO12 = 40%RWH-Quinoa; DMO13 = 60%RWH-Corn; DMO14 = 60%RWH-Soybeans; DMO15 = 60%RWH-Wheat; DMO16 = 60%RWH-Quinoa; DMO17 = 80%RWH-Corn; DMO18 = 80%RWH-Soybeans; DMO19 = 80%RWH-Wheat; DMO20 = 80%RWH-Quinoa; DMO21 = 100%RWH-Corn; DMO22 = 100%RWH-Soybeans; DMO23 = 100%RWH-Wheat; DMO24 = 100%RWH-Quinoa.

**Fig 6:**
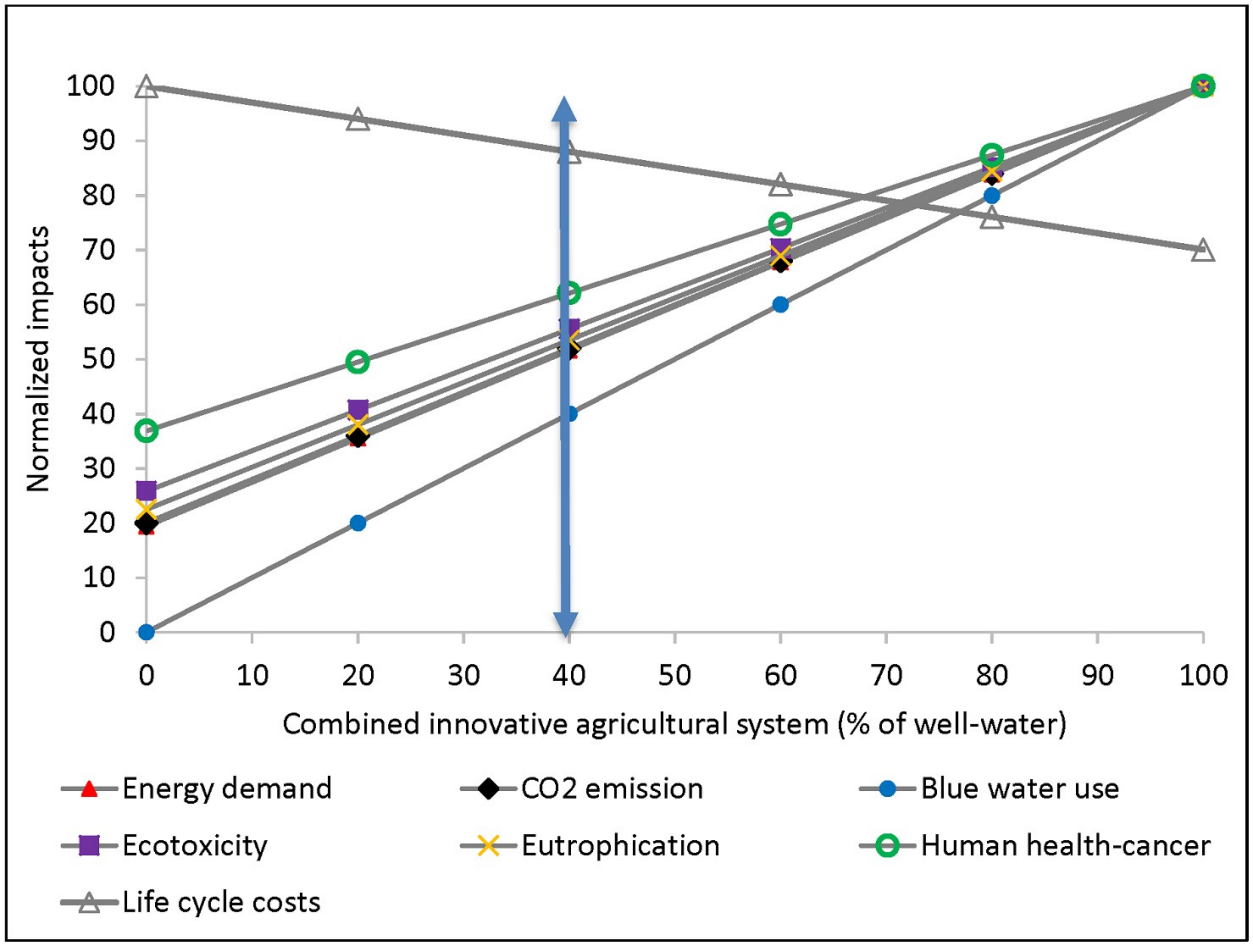
Sensitivity of sustainability indicators of combined systems (combined optimal agricultural RWH and well-water system) to the fraction of well-water; vertical arrow indicates an example threshold 40%; the % may be adjusted lower or higher. Note: The LCIA and LCCA values for each category were normalized with respect to maximum value.

For Group 2 DMOs, the functional unit LCIA values varied with the representative crop types (the ∝-parameter) and well-water fraction of the combined system of RWH and well-water systems (Fig 5a; and S1 Supporting Information). Importantly, LCIA values of all Group 2 DMOs were lower than well-water irrigation, mainly due to the optimal system with no pump and no holding tank. The mean-normalized energy demand was lowest for DMO 24 at 0.20, with no well-water support (100% RWH-Quinoa), and highest for DMO 1 at 2.08, with all well-water irrigation (0% RWH- Corn), due to the ∝-parameter (0.49 versus 1.00) and % rainwater use. This was also explained by combined systems’ impact sensitivity analyses (Fig 6): a hypothetical optimal agricultural RWH system with a fraction of well-water support at 0.4 (0.6 RWH:0.4 Well water) reduced human health-cancer impact by 38%, energy demand by 48%, and blue water use by 60%, with respect to well-water irrigation. The holistic sustainability scores revealed DMO 24, 23, 22, and 20 were the most sustainable, with scores of 1.00, 0.58, 0.56, and 0.55, respectively. DMO 21 (irrigating corn without well-water support) had a lower sustainability score, 0.49, than the quinoa with well-water support, a fraction of 0.2 (Fig 5b).

The life cycle cost of optimal RWH was higher—$0.06/m^3^—than well-water irrigation at $0.04/m^3^, which varied with the fraction of well-water support. Note that the life cycle costs are the costs of installing, replacing, operating and maintaining the system (see S1 Supporting Information for details); however, Life cycle cumulative energy cost savings occur due to the reductions of cumulative energy demand LCIA impact category. Comparing with higher irrigation water prices, e.g., an average irrigation water price of $0.1/m^3^ [70] these differences can be recouped by net savings in LCIA values such as life cycle cumulative energy cost savings—details on calculating energy cost savings can be found elsewhere [55].

### 3.3 Basin-wide sustainability indicators of agricultural systems

**Fig 7:**
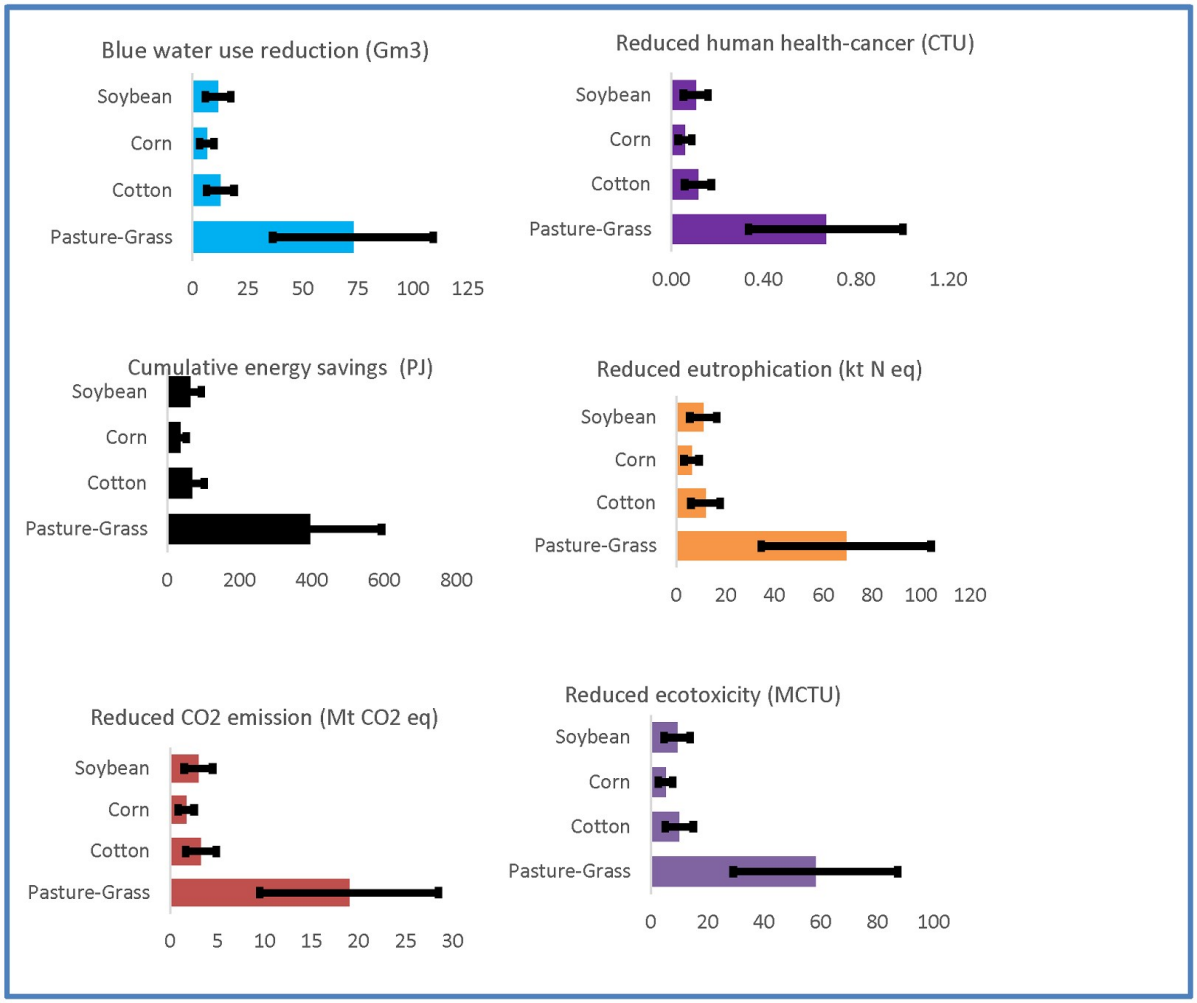
Sustainability indicators of agricultural systems of four RWH design configurations to irrigate the basin’s four major crops basin wide at a 25% adoption rate; results correspond to the most sustainable DMOs for each crop type with the variation in supplemental water demands of ±50% as depicted by horizontal bars. Unit prefixes are P = Peta; M = Mega; t = Metric ton; k = kilo; G = Giga; and CTU = comparative toxic units.

The scaling of functional unit sustainability indicators to basin scale at a reasonable adoption rate of 25% RWH provided broader perspectives into sustainability tradeoffs. Sustainability indicators, in terms of net LCIA benefits or savings of DMOs with respect to well-water irrigation (as defined in Eq 11), were higher for the larger volumetric well-water offset that also related to irrigational requirements and crop acreage. DMO 12 (no-pump system with polyethylene tank soybeans irrigation)—among Group 1’s most sustainable DMOs (9, 10, 11, 12) irrigating four crops in the basin—was the most sustainable option (holistic sustainability score of 1.00). Yet basin-wide net benefits were higher for DMO 9 (no pump system with PE tank pasture-grass irrigation) due to greater crop area and volumetric well-water offset. The average net LCIA savings due to 25% RWH adoption basin wide for pasture-grass irrigation (DMO 9) were cumulative energy savings at 400 Peta Joule (=10^15^ J) and reductions in CO_2_ emission, blue water use, ecotoxicity, eutrophication, and human health-cancer impacts at 19 Mt CO_2_ eq., 73 Gm^3^, 58 MCTU (CTU = Comparative Toxic Units), 69 kt N eq., and 0.7 CTU, respectively (Fig 7). Relative net benefits due to a 25% RWH adoption for four major crops irrigations (i.e., net LCIA benefit/total benefits of RWH) ranged from 70% pasture-grass, to 12% soybeans, to 6% cotton and 11% corn.

Because pasture-grass has the highest supplemental water needs at 0.39 m/y and largest crop area of 8,063 km^2^ it has a potential to improve sustainability (larger benefits) by offsetting larger volumetric well-water irrigation. Potential benefits of pasture-grass support the ideas of pasture cropping—pasture cropping refers to planting annual cereal crops into living perennial pasture, and permaculture which is a permanent, regenerative agriculture practice with potential to reduce and even reverse the environmental impacts associated with grain production and improving hydrology and ecosystem sustainability [71]. Pasture cropping has made significant contributions to sustainable land management in Australia and has the potential for wider adoption across the world.

**Fig 8:**
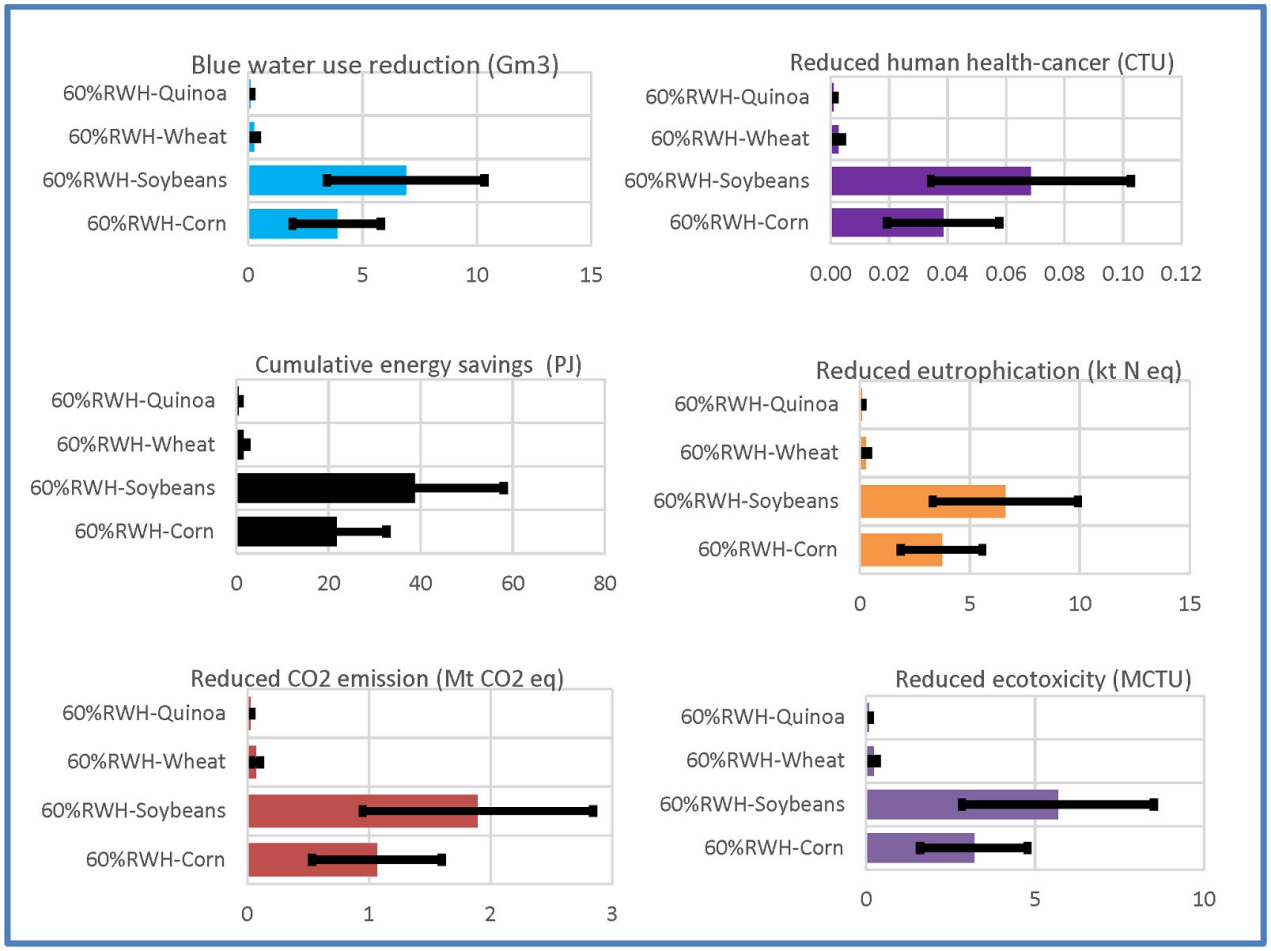
Sustainability indicators of agricultural systems of combined RWH and well-water systems at 0.4 well-water:0.6RWH to irrigate the four globally representative crops at a 25% adoption rate basin wide; results correspond to the most sustainable DMOs for each crop type with the variation in supplemental water demands of ±50% as depicted by horizontal bars. Unit prefixes are P = Peta; M = Mega; t = Metric ton; k = kilo; G = Giga; and CTU = comparative toxic units.

When adopted to basin scale rate of 25%, the relative benefits (i.e., net LCIA benefit/total benefits of RWH) due to combined system adoption for four representative crops’ irrigation ranged from 62% soybeans, to 35% corn, to 2% wheat and 1% quinoa. For a hypothetical system supported with well-water at a fraction of 0.4 for soybean irrigation, the net average LCIA savings due to soybean irrigation ranged from cumulative energy at 39 Peta Joule (=10^15^ J) and reductions in CO_2_ emission, blue water use, ecotoxicity, eutrophication, and human health-cancer indicators at 1.9 Mt CO_2_ eq., 6.9 Gm^3^, 5.7 MCTU, 6.6 kt N eq., and 0.07 CTU, respectively (Fig 8). The benefits were the greatest due to soybeans irrigation due primarily to larger crop acreage and volumetric well-water replacement. S1 Supporting Information provides additional details. Note that these LCIA savings were based on currently cultivated representative crops (corn, soybeans, and wheat), and the quinoa’s area being equivalent to that of wheat’s. Benefits thus vary with crop acreage and utilization of fraction of rainwater with well-water support.

**Fig 9:**
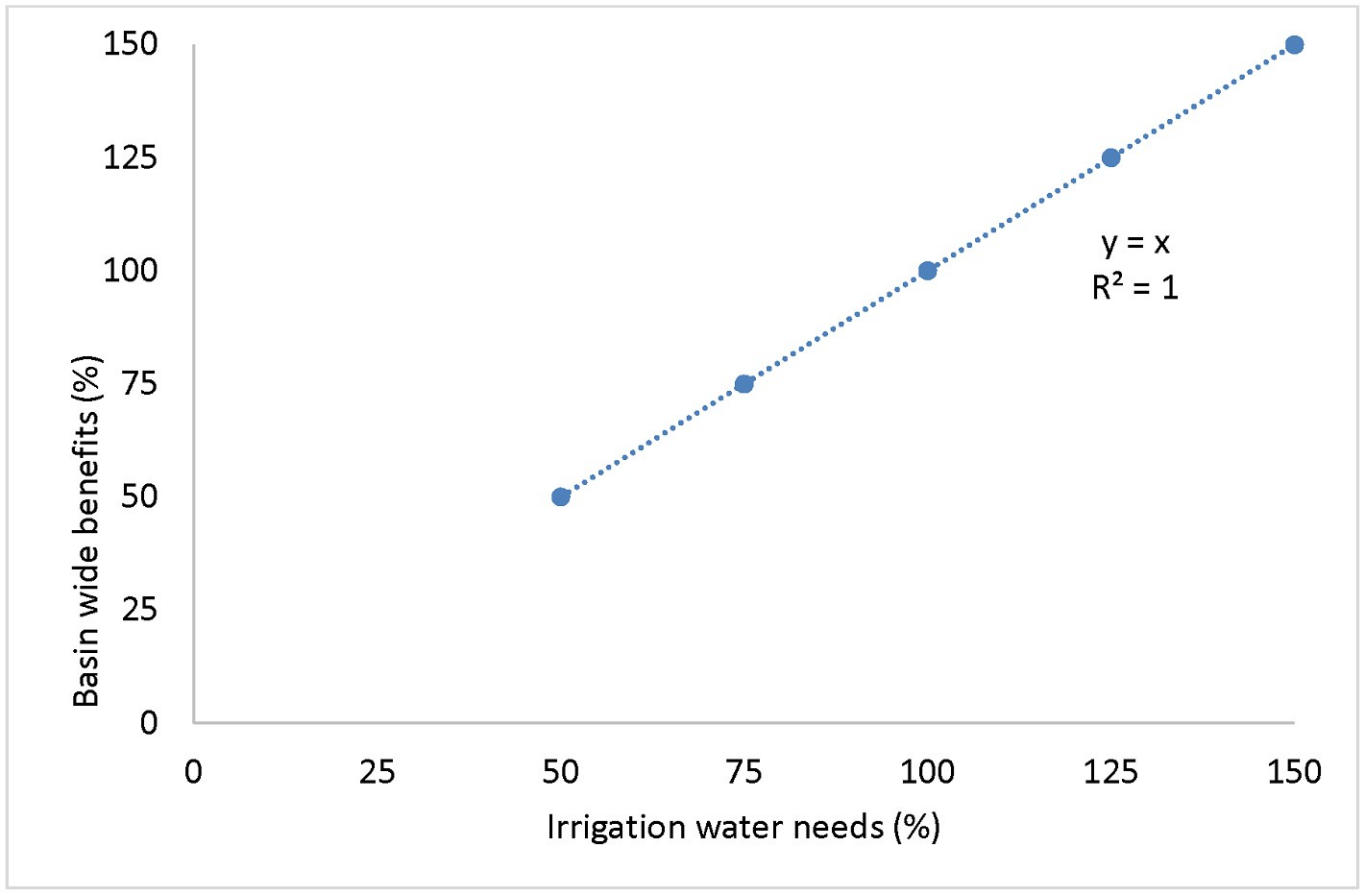
Sensitivity of sustainability indicators of combined systems (combined RWH and well-water systems) to crop irrigation water needs at a 25% adoption rate basin wide. Percentages (%) were computed with respect to original irrigation water needs of crops.

We addressed the sensitivity of basin-wide sustainability indicators in terms of net LCIA benefits (with respect to conventional well-water irrigation impacts) to scaling variables: adoption rates, service life, crop area, and irrigation water needs. Sensitivity analysis of the basin-wide sustainability indicators to each of the variables (adoption rates, service life, crop area, and irrigation water need) showed linear relationships, as expected from Eq 11. To illustrate this variation, the sensitivity of irrigation water needs at ±50% of actual irrigation water needs is reported in Fig 9 (also depicted by bars in Figs 7 and 8). Sensitivity analyses of basin-wide sustainability indicators of agricultural systems to adoption rates, service life, and crop area are provided in S1 Supporting Information.

These DMOs may be different for different geographic location, rainfall/runoff potential (or rainwater availability) with respect to seasonality (dry and wet conditions), crop types and water demand, systems operation and maintenance requirements, and material and energy infrastructure. As an example, tank size (capability of storage) varies with the location and rainwater harvesting potential (availability of rainwater). We attempted to include diverse design configurations of RWH as DMOs by categorizing them into two groups (design/material specific configurations and % RWH availability).

It is noted that the LCIA values are influenced by LCA model parameters, LCIA characterization methods, as well as information uncertainty (e.g., information availability, accuracy, reliability, or a certain degree of spatial and temporal variation) [72–76]; however, this was beyond the scope of current analysis.

## Summary and study implications

Using a comprehensive sustainability assessment approach to 40 decision management objectives (DMOs) of agricultural systems as applied to a southeastern U.S. basin, we addressed key scientific questions of agricultural sustainability posed at the beginning of this work. Our approach is general enough to be applied for the characterization of agricultural sustainability indicators in addition to evaluate sustainability of other green infrastructure configurations at varied locations in the face of water scarcity and crop irrigation needs of a growing population across the U.S. and the world. Amongst the 40 DMOs representing diverse agricultural systems, optimal RWH with no well-water support was found most sustainable option for globally representative crops irrigation (corn, soybeans, wheat, and quinoa). However, certain agricultural systems were found more sustainable than others—we reported most sustainable designs and corresponding sustainability indicators of basin scale agricultural systems at a reasonable adoption rate of 25%. The agricultural systems comprised of alternative crop types and irrigation practices of agricultural RWH combined with well-water irrigation system. Sixteen DMOs of Group 1 incorporated DMOs consisting RWH infrastructure, tank material, pump, and pumping energy for irrigating the major four crops (pasture-grass, soybeans, corn, and cotton) within the basin. Twenty-four DMOs of Group 2 incorporated DMOs of combined systems, a combination of an optimal agricultural RWH system with a support of well-water irrigating globally representative crops (quinoa, wheat, soybeans, and corn).

Environmental setting (e.g., crop types, crop cultivation acreage, irrigational practice) and design configurations (e.g., RWH system with/without pump and alternative materials) affected the DMO sustainability. From Group 1, DMOs 12, 11, 10, and 9 (i.e., no pump systems with PE tank for soybeans, corn, cotton, and pasture-grass irrigation) were the most sustainable, with holistic sustainability scores of 1.00, 0.88, 0.58, and 0.48, respectively. From Group 2, DMOs 24, 23, 22, and 20 (i.e., 0.00 Well water:1.00 RWH, with no tank no pump for quinoa, wheat, soybeans, and corn irrigation) resulted in scores of 1.00, 0.58, 0.56, and 0.49, respectively. It is noted that the ranking and the scores of the DMOs is limited by the number of DMOs and their configuration that may be different for different geographic location, rainfall/runoff potential, crop types and water demand, and system operation and maintenance requirements. The LCIA benefits would increase linearly with the proportion of the basin’s crop area allocated for RWH. We reported optimal results based on the most sustainable designs and corresponding sustainability indicators of basin scale agricultural systems at a reasonable adoption rate of 25%. At a basin-wide RWH adoption rate of 25%, the benefits relative to well-water of the most sustainable design configuration in Group 1 (i.e., DMO 9: no pump system with PE tank pasture-grass irrigation) provided cumulative energy savings of 395 Peta Joule and reductions in CO_2_ emission, blue water use, ecotoxicity, eutrophication, and human health-cancer at 19 Mt CO_2_ eq.,73 Gm^3^, 58 MCTU, 69 kt N eq., and 0.7 CTU, respectively.

Net environmental and human health benefits of basin-wide RWH at 25% adoption were higher for a crop with greater cultivation area and volumetric replacement of well-water. Comparative, relative net benefits due to RWH for pasture-grass, cotton, corn, and soybeans were 70%, 12%, 6% and 11%, respectively. The relative net benefits due to a 25% adoption rate of combined system (combined RWH with well-water) basin wide for four representative crops irrigations ranged from 62% soybeans, to 35% corn, to 2% wheat and 1% quinoa. Net LCIA savings were based on currently cultivated representative crops (corn, soybeans, and wheat) in the Albemarle-Pamlico basin; although quinoa was not currently cultivated, benefits for the area equivalent to wheat were reported for comparison.

Although the DMO sustainability varied with the environmental settings and design configurations, with appropriate design modifications and procurement of local data, our approach is transferrable to evaluate sustainability of other water management and green infrastructure practices in other locations. Modifications related to location-specific agricultural innovations may include re-design of RWH system components such as sedimentation chambers, pipe materials, energy use, and pivot center, as deemed necessary for site- and crop-specific requirements in addition to meeting the regulatory requirements (water quality/quantity). Other green infrastructure includes on-site graywater treatment and reuse, rain gardens, green roofs, permeable pavements, and/or a mix of these with conventional gray infrastructure (e.g., pipe network improvements, water detention structures, pump stations) for drainage and flooding problems such as in the North Atlantic coastal communities of New York [77] and across the Great Lakes basin [78], among others.

This work builds on a prior LCA study [30] that provided LCIA categories of four agricultural RWH configurations for reference crop (corn) irrigation, and a modified eco-efficiency framework [47]. Sustainability indicators were scaled with functional unit impacts, comparing conventional well-water irrigation supplies that were consistent with widely accepted LCA practice [55, 79]. Sensitivity analysis of the basin-wide sustainability indicators to the adoption rates, service life, crop area, and irrigation water needs was also addressed. Variations due to economies of scale arising from wider adoptions, spatially heterogeneous processes (e.g., mixed land cover), and temporally varying data were beyond the scope of this study. Such variations would be of particular interest when considering hydrologic indicators at the watershed scale [54]; however, that was not the case for the current study. It is also noted that consumer/human behavior on water withdrawal/consumption, and crop benefit, or crop value (such as market value and nutritional benefit) could serve as additional sustainability indicators but these were beyond the scope of current study.

These results can target RWH crop irrigation, improving agricultural sustainability in the southeastern U.S. and beyond [56]. A holistic sustainability score provided an overall sustainability status to enable a decision regarding the selection of a DMO (i.e., a sustainable agricultural innovation). Individual indicators provided insights into tradeoffs, which can inform selection of a particular DMO with an emphasis on indicator of choice. Here, tradeoffs imply to the comparative basin scale RWH sustainability in terms of net LCIA benefits. The results also have broader implications in the context of globally prioritized crops in food-insecure regions (Asia, Africa, South-and Central America) in the face of a growing world population, and food and water requirements [5].

Water resource planners and analysts can use these results to prioritize RWH crop irrigation, informing decisions on sustainable cropping at local farms and cooperatives as well as organic crop farming practices [80]. Our approach provided two ways of selecting a DMO: (1) using an integrated sustainability score (2) using the disparate basin-wide sustainability indicators. An analyst can use an integrated sustainability indicator to select a most sustainable DMO, or they can use one or more of these disparate indicators depending upon their impact priority. For example, an analyst would choose DMO12 with scores of 1.00 (i.e., no pump systems with PE tank for soybeans irrigation) from Group 1. They could also choose DMO24 with scores of 1.00 (i.e., 100%RWH-Quinoa) from Group 2. Further, the disparate indicators at the basin scale provided insights into relative benefits of a DMO with respect to well-water irrigation by considering the variables such as adoption rates, system service life, crop area, and irrigation water needs. In addition, our approach of estimating irrigation water needs using the ∝-parameter (i.e., actual crop water need to reference crop (corn) water need ratio) is applicable to various climatic regions since it normalizes the change in water needs. This study demonstrated a flexible and generally applicable assessment methodology to assess the sustainability of various agricultural practices. An important next step is to engage stakeholders with local knowledge regarding implementation and possible data from field trials. The designs may be further enhanced by considering climate-smart water management applications (e.g., Internet of Things) [81, 82]. This includes integrating the latest innovative Global Positioning System technologies, inexpensive monitoring devices, wireless technologies, as well as available cloud data centers to precisely account for water needs and losses during crop irrigation. Increased crop yields, decreased water demand, and reduced environmental impacts each contribute to environmental sustainability. Government-private partnerships combined with increased collaboration amongst stakeholders—including local farmers, agronomists, economists, and RWH practitioners—play an important role moving forward.

## Supporting information

S1 Supporting Information (DOCX)
