# Supplementary material for: Sustainability assessment of agricultural rainwater harvesting: Evaluation of alternative crop types and irrigation practices
Source: PLoS One. 2019 May 10;14(5):e0216452. doi: 10.1371/journal.pone.0216452 (PMC6510416; doi:10.1371/journal.pone.0216452)
Supplement: S1 Supporting Information — (DOCX) [file pone.0216452.s001.docx]

**Supplementary Information (SI) for**

**Sustainability assessment of agricultural rainwater harvesting: evaluation of alternative crop types and irrigation practices**

Santosh R. Ghimire^1⁋*^, John M. Johnston^2⁋*^

^1^Global Sustainability and Life Cycle Consultant, LLC

U.S. Environmental Protection Agency, Office of Research and Development, Computational Exposure Division, Athens, Georgia, USA

ORCID: [0000-0001-7038-4167](https://orcid.org/0000-0001-7038-4167)

^2^U.S. Environmental Protection Agency, Office of Research and Development, Computational Exposure Division, Athens, Georgia, USA

ORCID: [0000-0002-5886-7876](https://orcid.org/0000-0002-5886-7876)

^1^Current Address: Global Sustainability and Life Cycle Consultant, LLC, Athens, Georgia, USA

^*^Corresponding author

E-mail: [sghimire02@gmail.com](mailto:sghimire02@gmail.com) (SRG); [Johnston.JohnM@epa.gov](mailto:Johnston.JohnM@epa.gov) (JMJ)

^⁋^These authors contributed equally to this work.

Contents

[SI1: LCIA and LCCA of Group 1 and Group 2 DMOs 3](#_Toc7257109)

[Table S1. Group 1 DMOs’ LCIA and LCCA values. 4](#_Toc7257110)

[Table S2. Description of LCCA of four agricultural RWH configurations used in Group 1 DMOs [2]. 5](#_Toc7257111)

[Table S3. LCIA values of well-water irrigation systems comparable to Group 1 DMOs. 7](#_Toc7257112)

[Table S4. Description of LCCA of optimal agricultural RWH system used in Group 2 DMOs [2]. 9](#_Toc7257113)

[Table S5. Description of LCCA of well-water irrigation system used in Group 2 DMOs [2]. 10](#_Toc7257114)

[Table S6. Group 2 DMOs’ LCIA and LCCA values. 11](#_Toc7257115)

[Table S7. LCIA values of well-water irrigation systems comparable to Group 2 DMOs. 12](#_Toc7257116)

[SI2: DEA for holistic sustainability analysis 17](#_Toc7257117)

[Table S8. Example matrix of DMO versus weights (*w_i_*) for DEA. 18](#_Toc7257118)

[Table S9. Mean-normalized LCIA and LCCA data set. 19](#_Toc7257119)

[SI3: Basin-wide RWH sustainability indicators 24](#_Toc7257120)

[Table S10. Net LCIA benefits of combined systems in Group 2. 24](#_Toc7257121)

[Fig S1. Sensitivity of sustainability indicators of combined systems. 25](#_Toc7257122)

[Fig S2. Sensitivity of sustainability indicators of RWH-Quinoa system to quinoa crop area. 25](#_Toc7257123)

[Fig S3. Sensitivity of sustainability indicators of combined systems to system service life. 26](#_Toc7257124)

[REFERENCES 26](#_Toc7257125)

# **SI1: LCIA and LCCA** **of Group 1 and Group 2 DMOs**

**Group 1 DMOs:** Life cycle impact assessment (LCIA) category values and life cycle cost assessment (LCCA) present values per functional unit of 1m^3^ of rainwater delivery, pertinent to the Group 1 decision management objectives (DMOs), are summarized in Tables S1 and S1. LCIA values of the four configurations of agricultural RWH for reference crop, corn irrigation were adopted from [Ghimire, Johnston [1]](#_ENREF_1). LCCA values of the four configurations were calculated consistent with [Ghimire and Johnston [2]](#_ENREF_2) . LCIAs of well-water irrigation systems comparable to each of the 16 DMOs are provided in Table S3.

Note that the Baseline System (Tables S1 and S2) consisted of the agricultural RWH system for reference crop, corn irrigation with the following components: 13000 m^3^ sediment chamber, 155 m 101.6 mm diameter collection and distribution polyvinyl chloride (PVC) pipe, a polyethylene (PE) water-holding Tank, a pump, pumping energy at 0.30 kWh/m^3^, a pivot-center, control valves, and check valves, adopted from [Ghimire, Johnston [1]](#_ENREF_1). All DMOs were modifications of the Baseline System.

## **Table S1. Group 1 DMOs’ LCIA and LCCA values.**

| Description | DMO | Energy demand (MJ/m^3^) | CO_2_ emission (kg CO2 eq/m^3^) | Blue water use (m^3^/m^3^) | Ecotoxicity (CTU/m^3^) | Eutrophication (kg N eq/m^3^) | Human health- cancer (CTU/m^3^) | Life cycle costs present value ($/m^3^) |
| --- | --- | --- | --- | --- | --- | --- | --- | --- |
| Baseline System Pasture-Grass irrigation | DMO1 | 1.01E+01 | 5.89E-01 | 2.39E-03 | 9.27E-04 | 2.40E-03 | 2.53E-11 | 0.24 |
| Baseline System Cotton irrigation | DMO2 | 8.43E+00 | 4.91E-01 | 1.99E-03 | 7.73E-04 | 2.00E-03 | 2.11E-11 | 0.20 |
| Baseline System Corn (Reference crop) irrigation | DMO3 | 5.48E+00 | 3.19E-01 | 1.29E-03 | 5.02E-04 | 1.30E-03 | 1.37E-11 | 0.13 |
| Baseline System Soybean irrigation | DMO4 | 4.85E+00 | 2.82E-01 | 1.14E-03 | 4.44E-04 | 1.15E-03 | 1.21E-11 | 0.12 |
| Concrete Tank System Pasture-Grass irrigation | DMO5 | 9.98E+00 | 5.85E-01 | 2.65E-03 | 9.05E-04 | 2.43E-03 | 2.55E-11 | 0.26 |
| Concrete Tank System Cotton irrigation | DMO6 | 8.32E+00 | 4.87E-01 | 2.21E-03 | 7.54E-04 | 2.03E-03 | 2.12E-11 | 0.22 |
| Concrete Tank System Corn irrigation | DMO7 | 5.41E+00 | 3.17E-01 | 1.44E-03 | 4.90E-04 | 1.32E-03 | 1.38E-11 | 0.14 |
| Concrete Tank System Soybean irrigation | DMO8 | 4.78E+00 | 2.80E-01 | 1.27E-03 | 4.34E-04 | 1.17E-03 | 1.22E-11 | 0.13 |
| No pump System PE Tank Pasture-Grass irrigation | DMO9 | 2.88E+00 | 1.54E-01 | 1.10E-03 | 5.79E-04 | 5.30E-04 | 1.21E-11 | 0.21 |
| No pump System PE Tank Cotton irrigation | DMO10 | 2.40E+00 | 1.29E-01 | 9.14E-04 | 4.82E-04 | 4.42E-04 | 1.01E-11 | 0.17 |
| No pump System PE Tank Corn irrigation | DMO11 | 1.56E+00 | 8.36E-02 | 5.94E-04 | 3.13E-04 | 2.87E-04 | 6.58E-12 | 0.11 |
| No pump System PE Tank Soybean irrigation | DMO12 | 1.38E+00 | 7.40E-02 | 5.26E-04 | 2.77E-04 | 2.54E-04 | 5.82E-12 | 0.10 |
| No pump System Concrete Tank Pasture-Grass irrigation | DMO13 | 2.74E+00 | 1.50E-01 | 1.36E-03 | 5.57E-04 | 5.60E-04 | 1.23E-11 | 0.23 |
| No pump System Concrete Tank Cotton irrigation | DMO14 | 2.28E+00 | 1.25E-01 | 1.14E-03 | 4.64E-04 | 4.66E-04 | 1.02E-11 | 0.19 |
| No pump System Concrete Tank Corn irrigation | DMO15 | 1.49E+00 | 8.14E-02 | 7.39E-04 | 3.02E-04 | 3.03E-04 | 6.65E-12 | 0.12 |
| No pump System with concrete Tank Soybean irrigation | DMO16 | 1.31E+00 | 7.20E-02 | 6.54E-04 | 2.67E-04 | 2.68E-04 | 5.88E-12 | 0.11 |

## **Table S2.** **Description of LCCA of four agricultural RWH configurations used in Group 1 DMOs [**[**2**](#_ENREF_2)**].**

| Configuration 🡪 | Configuration 1: Baseline System | | Configuration 2: Concrete Tank System | | Configuration 3: No pump System PE Tank | | Configuration 4: No pump System Concrete Tank | | Reference |
| --- | --- | --- | --- | --- | --- | --- | --- | --- | --- |
| System description | Cost, base date (2014 $ value) | 50-yr present value, $ | Cost, base date (2014 $ value) | 50-yr present value, $ | Cost, base date (2014 $ value) | 50-yr present value, $ | Cost, base date (2014 $ value) | 50-yr present value, $ | Not applicable |
| Sediment chamber, 13000 m^3^ @ $3.8/m^3^ | 49,387 | 49,387 | 49,387 | 49,387 | 49,387 | 49,387 | 49,387 | 49,387 | [[1](#_ENREF_1), [3](#_ENREF_3)] |
| Collection & distribution pipe, 155 m (PVC 101.6 mm dia.) @ $15.1/m | 2,339 | 2,339 | 2,339 | 2,339 | 2,339 | 2,339 | 2,339 | 2,339 | [[1](#_ENREF_1), [4](#_ENREF_4)] |
| Tank 1 unit, 606 m^3^ | 180,532 | 180,532 | 212,878 | 212,878 | 180,532 | 180,532 | 212,878 | 212,878 | [[5](#_ENREF_5)] |
| Pump, 1 unit | 3,934 | 3,934 | 3,934 | 3,934 | - | - | - | - | [[6](#_ENREF_6)] |
| Pivot-center, 1 unit | 38,296 | 38,296 | 38,296 | 38,296 | 38,296 | 38,296 | 38,296 | 38,296 | [[7](#_ENREF_7)] |
| Control valve, 4 in, cast iron, 1 piece | 164 | 164 | 164 | 164 | 164 | 164 | 164 | 164 | [[8](#_ENREF_8)] |
| Check valve, 4 in, cast iron, 1 piece | 596 | 596 | 596 | 596 | 596 | 596 | 596 | 596 | [[8](#_ENREF_8)] |
| Investment sub-total, I | 275,248 | 275,248 | 307,594 | 307,594 | 271,314 | 271,314 | 303,660 | 303,660 | As compiled |
| Pumps, 3 units (replacements occur at the end of 15, 30, and 45 years) | 30,555 | 6,970 | 30,555 | 6,970 | - | - | - | - | As compiled |
| Pivot-center, 2 units (replacements occur at the end of 20 and 40 years) | 194,090 | 44,273 | 194,090 | 44,273 | 194,090 | 44,273 | 194,090 | 44,273 | As compiled |
| Check valve, 4 in, cast iron, 6 pieces (replacements occur at the end of 7.5, 15, 22.5, 30, 37.5, and 45 years) | 2,300 | 525 | 2,300 | 525 | 2,300 | 525 | 2,300 | 525 | As compiled |
| Control valve, 4 in, cast iron, 6 pieces (replacement occur at the end of 7.5, 15, 22.5, 30, 37.5, and 45 years) | 8,338 | 1,902 | 8,338 | 1,902 | 8,338 | 1,902 | 8,338 | 1,902 | As compiled |
| Replacement sub-total, R | 235,283 | 53,670 | 235,283 | 53,670 | 204,728 | 46,700 | 204,728 | 46,700 | As compiled |
| Pump, (10/15 of a unit) | 11,498 | 2,623 | 11,498 | 2,623 | - | - | - | - | [[9](#_ENREF_9)] |
| Pivot-center, 10/20 unit | 83,943 | 19,148 | 83,943 | 19,148 | 83,943 | 19,148 | 83,943 | 19,148 | [[9](#_ENREF_9)] |
| Check valve, 4 in, cast iron, 2.5/7.5 piece | 240 | 55 | 240 | 55 | 240 | 55 | 240 | 55 | [[9](#_ENREF_9)] |
| Control valve, 4 in, cast iron, 2.5/7.5 piece | 871 | 199 | 871 | 199 | 871 | 199 | 871 | 199 | [[9](#_ENREF_9)] |
| Residuals value sub-total, V | 96,552 | 22,024 | 96,552 | 22,024 | 85,054 | 19,401 | 85,054 | 19,401 | As compiled |
| Dredging, 7108 m^2^ by 0.02 m depth/yr @ $20/m^3^ | 2,843 | 73,155 | 2,843 | 73,155 | 2,843 | 73,155 | 2,843 | 73,155 | [[10-12](#_ENREF_10)] |
| Sediment disposal cost @ $7/m^3^ | 995 | 25,604 | 995 | 25,604 | 995 | 25,604 | 995 | 25,604 | [[10-12](#_ENREF_10)] |
| O &M (1.7% of Investment) | 4,679 | 120,395 | 5,229 | 134,544 | 4,612 | 118,674 | 5,162 | 132,823 | [[13](#_ENREF_13)] |
| Pumping energy cost, 0.3 kWh/m^3^ @ $0.103/kWh | 2,809 | 72,270 | 2,809 | 72,270 | - | - | - | - | [[1](#_ENREF_1)] |
| Annual costs sub-total, A | 11,326 | 291,424 | 11,876 | 305,573 | 8,451 | 217,433 | 9,001 | 231,582 | As compiled |
| Life cycle cost (Lc) = I + R - V + A | 425,305 | 598,317 | 458,201 | 644,813 | 399,438 | 516,045 | 432,335 | 562,541 | As compiled |
| $/m^3^ | 0.09 | 0.13 | 0.10 | 0.14 | 0.09 | 0.11 | 0.10 | 0.12 |  |

## **Table S3. LCIA values of well-water irrigation systems comparable to Group 1 DMOs.**

| Comparable DMO | Energy demand (MJ/m^3^) | CO_2_ emission (kg CO2 eq/m^3^) | Blue water use (m^3^/m^3^) | Ecotoxicity (CTU/m^3^) | Eutrophication (kg N eq/m^3^) | Human health-cancer (CTU/m^3^) |
| --- | --- | --- | --- | --- | --- | --- |
| DMO1 | 1.29E+01 | 6.37E-01 | 1.85E+00 | 2.06E-03 | 2.29E-03 | 2.91E-11 |
| DMO2 | 1.07E+01 | 5.31E-01 | 1.54E+00 | 1.71E-03 | 1.91E-03 | 2.43E-11 |
| DMO3 | 6.99E+00 | 3.45E-01 | 1.00E+00 | 1.11E-03 | 1.24E-03 | 1.58E-11 |
| DMO4 | 6.18E+00 | 3.05E-01 | 8.86E-01 | 9.85E-04 | 1.10E-03 | 1.40E-11 |
| DMO5 | 1.29E+01 | 6.37E-01 | 1.85E+00 | 2.06E-03 | 2.29E-03 | 2.91E-11 |
| DMO6 | 1.07E+01 | 5.31E-01 | 1.54E+00 | 1.71E-03 | 1.91E-03 | 2.43E-11 |
| DMO7 | 6.99E+00 | 3.45E-01 | 1.00E+00 | 1.11E-03 | 1.24E-03 | 1.58E-11 |
| DMO8 | 6.18E+00 | 3.05E-01 | 8.86E-01 | 9.85E-04 | 1.10E-03 | 1.40E-11 |
| DMO9 | 1.29E+01 | 6.37E-01 | 1.85E+00 | 2.06E-03 | 2.29E-03 | 2.91E-11 |
| DMO10 | 1.07E+01 | 5.31E-01 | 1.54E+00 | 1.71E-03 | 1.91E-03 | 2.43E-11 |
| DMO11 | 6.99E+00 | 3.45E-01 | 1.00E+00 | 1.11E-03 | 1.24E-03 | 1.58E-11 |
| DMO12 | 6.18E+00 | 3.05E-01 | 8.86E-01 | 9.85E-04 | 1.10E-03 | 1.40E-11 |
| DMO13 | 1.29E+01 | 6.37E-01 | 1.85E+00 | 2.06E-03 | 2.29E-03 | 2.91E-11 |
| DMO14 | 1.07E+01 | 5.31E-01 | 1.54E+00 | 1.71E-03 | 1.91E-03 | 2.43E-11 |
| DMO15 | 6.99E+00 | 3.45E-01 | 1.00E+00 | 1.11E-03 | 1.24E-03 | 1.58E-11 |
| DMO16 | 6.18E+00 | 3.05E-01 | 8.86E-01 | 9.85E-04 | 1.10E-03 | 1.40E-11 |

**Group 2 DMOs:** LCIA and LCCA values per functional unit of 1m^3^ of rainwater delivery, pertinent to Group 2 DMOs, are summarized in Tables S4-S6, and comparable well-water DMOs’ LCIAs are summarized in Table S7.

## **Table S4. Description of LCCA of optimal agricultural RWH system used in Group 2 DMOs [**[**2**](#_ENREF_2)**].**

| Description | Cost, base date  (2014 $ value) | 50-yr present value, $ |
| --- | --- | --- |
| Sediment chamber, 13000 m^3^ @ $3.8/m^3^ | 49,387 | 49,387 |
| Collection & distribution pipe, 155 m (PVC 101.6 mm dia.) @ $15.1/m | 2,339 | 2,339 |
| Pivot-center, 1 unit | 38,296 | 38,296 |
| Control valve, 4", cast iron, 1 piece | 164 | 164 |
| Check valve, 4 ", cast iron, 1 piece | 596 | 596 |
| Investment sub-total, I | 90,782 | 90,782 |
| Pivot-center, 2 units (replacements occur at the end of 20 and 40 years) | 194,090 | 44,273 |
| Check valve, 4 ", cast iron, 6 pieces (replacements occur at the end of 7.5, 15, 22.5, 30, 37.5, and 45 years) | 2,300 | 525 |
| Control valve, 4", cast iron, 6 pieces (replacement occur at the end of 7.5, 15, 22.5, 30, 37.5, and 45 years) | 8,338 | 1,902 |
| Replacement sub-total, R | 204,728 | 46,700 |
| Pivot-center, 10/20 unit | 83,943 | 19,148 |
| Check valve, 4 ", cast iron, 2.5/7.5 piece | 240 | 55 |
| Control valve, 4", cast iron, 2.5/7.5 piece | 871 | 199 |
| Residuals value sub-total, V | 85,054 | 19,401 |
| Dredging, 7108 m^2^ by 0.02 m depth/yr @ $20/m^3^ | 2,843 | 73,155 |
| Sediment disposal cost @ $7/m^3^ | 995 | 25,604 |
| O &M (1.7% of Investment) | 1,543 | 39,709 |
| Annual costs sub-total, A | 5,382 | 138,468 |
| Life cycle cost (Lc) = I + R - V + A | 215,837 | 256,548 |
| Life cycle price of rainwater ($/m^3^) = Lc/life time rainwater supply | N/A | 0.06 |

## **Table S5. Description of LCCA of well-water irrigation system used in Group 2 DMOs [**[**2**](#_ENREF_2)**].**

| Description | Cost, base date (2014 $ value) | Discount rate (decimal) | 50-yr present value, $ | Reference |
| --- | --- | --- | --- | --- |
| Well, 1 unit | 7,500 | n/a | 7500.00 | USDA (2009) |
| Main pipeline (suction and discharge), 155 m (PVC 101.6 mm dia.) @ $15.1/m | 2,339 | n/a | 2,339 | [[1](#_ENREF_1), [4](#_ENREF_4)] |
| Pump, 1 unit | 3,934 | n/a | 3,934 | [[6](#_ENREF_6)] |
| Pivot-center, 1 unit irrigating 34 ha | 38,296 | n/a | 38,296 | [[7](#_ENREF_7)] |
| Control valve, 4", cast iron, 1 piece | 164 | n/a | 164 | [[8](#_ENREF_8)] |
| Check valve, 4 ", cast iron, 1 piece | 596 | n/a | 596 | [[8](#_ENREF_8)] |
| Investment sub-total, I | 52,829 | n/a | 52,829 | As compiled |
| Pumps, 3 units (replacements occur at the end of 15, 30, and 45 years) | 30,555 | 0.03 | 6,970 | As compiled |
| Pivot-center, 2 units (replacements occur at the end of 20 and 40 years) | 194,090 | 0.03 | 44,273 | As compiled |
| Check valve, 4 ", cast iron, 6 pieces (replacements occur at the end of 7.5, 15, 22.5, 30, 37.5, and 45 years) | 2,300 | 0.03 | 525 | As compiled |
| Control valve, 4", cast iron, 6 pieces (replacement occur at the end of 7.5, 15, 22.5, 30, 37.5, and 45 years) | 8,338 | 0.03 | 1,902 | As compiled |
| Replacement sub-total, R | 235,283 | n/a | 53,670 | As compiled |
| Pump, (10/15 of a unit) | 11,498 | 0.03 | 2,623 | [[9](#_ENREF_9)] |
| Pivot-center, 10/20 unit | 83,943 | 0.03 | 19,148 | [[9](#_ENREF_9)] |
| Check valve, 4 ", cast iron, 2.5/7.5 piece | 240 | 0.03 | 55 | [[9](#_ENREF_9)] |
| Control valve, 4", cast iron, 2.5/7.5 piece | 871 | 0.03 | 199 | [[9](#_ENREF_9)] |
| Residuals value sub-total, V | 96,552 | n/a | 22,024 | As compiled |
| O &M (1.7% of Investment) | 898 | 0.03 | 23,108 | [[13](#_ENREF_13)] |
| Pumping energy cost, 0.3 kWh/m^3^ @ $0.103/kWh | 2,809 | 0.03 | 72,270 | [[1](#_ENREF_1)] |
| Annual costs sub-total, A | 3,707 |  | 95,378 | As compiled |
| Life cycle cost (Lc) = I + R - V + A | 195,267 |  | 179,852 | As compiled |
| Life cycle price of rainwater ($/m^3^) = Lc/life time rainwater supply | N/A | N/A | 0.040 | As compiled |

## **Table S6. Group 2 DMOs’ LCIA and LCCA values.**

| Description of DMOs | DMO | Energy demand (MJ/m^3^) | CO_2_ emission (kg CO2 eq/m^3^) | Blue water use (m^3^/m^3^) | Ecotoxicity (CTU/m^3^) | Eutrophication (kg N eq/m^3^) | Human health- cancer (CTU/m^3^) | Life cycle costs ($/m^3^) |
| --- | --- | --- | --- | --- | --- | --- | --- | --- |
| 0%RWH-Corn | DMO1 | 6.99E+00 | 3.45E-01 | 1.00E+00 | 1.11E-03 | 1.24E-03 | 1.58E-11 | 0.04 |
| 0%RWH-Soybeans | DMO2 | 6.15E+00 | 3.03E-01 | 8.81E-01 | 9.80E-04 | 1.09E-03 | 1.39E-11 | 0.03 |
| 0%RWH-Wheat | DMO3 | 5.94E+00 | 2.93E-01 | 8.51E-01 | 9.46E-04 | 1.05E-03 | 1.34E-11 | 0.03 |
| 0%RWH-Quinoa | DMO4 | 3.42E+00 | 1.69E-01 | 4.91E-01 | 5.45E-04 | 6.08E-04 | 7.73E-12 | 0.02 |
| 20%RWH-Corn | DMO5 | 5.86E+00 | 2.90E-01 | 8.01E-01 | 9.48E-04 | 1.05E-03 | 1.38E-11 | 0.04 |
| 20%RWH-Soybeans | DMO6 | 5.16E+00 | 2.55E-01 | 7.05E-01 | 8.34E-04 | 9.23E-04 | 1.21E-11 | 0.04 |
| 20%RWH-Wheat | DMO7 | 4.98E+00 | 2.46E-01 | 6.81E-01 | 8.06E-04 | 8.91E-04 | 1.17E-11 | 0.04 |
| 20%RWH-Quinoa | DMO8 | 2.87E+00 | 1.42E-01 | 3.93E-01 | 4.65E-04 | 5.14E-04 | 6.75E-12 | 0.02 |
| 40%RWH-Corn | DMO9 | 4.74E+00 | 2.35E-01 | 6.01E-01 | 7.83E-04 | 8.56E-04 | 1.18E-11 | 0.05 |
| 40%RWH-Soybeans | DMO10 | 4.17E+00 | 2.06E-01 | 5.29E-01 | 6.89E-04 | 7.54E-04 | 1.04E-11 | 0.04 |
| 40%RWH-Wheat | DMO11 | 4.03E+00 | 1.99E-01 | 5.11E-01 | 6.66E-04 | 7.28E-04 | 1.00E-11 | 0.04 |
| 40%RWH-Quinoa | DMO12 | 2.32E+00 | 1.15E-01 | 2.95E-01 | 3.84E-04 | 4.20E-04 | 5.78E-12 | 0.02 |
| 60%RWH-Corn | DMO13 | 3.61E+00 | 1.79E-01 | 4.01E-01 | 6.18E-04 | 6.64E-04 | 9.80E-12 | 0.05 |
| 60%RWH-Soybeans | DMO14 | 3.18E+00 | 1.58E-01 | 3.53E-01 | 5.44E-04 | 5.84E-04 | 8.62E-12 | 0.04 |
| 60%RWH-Wheat | DMO15 | 3.07E+00 | 1.53E-01 | 3.41E-01 | 5.25E-04 | 5.65E-04 | 8.33E-12 | 0.04 |
| 60%RWH-Quinoa | DMO16 | 1.77E+00 | 8.79E-02 | 1.96E-01 | 3.03E-04 | 3.25E-04 | 4.80E-12 | 0.02 |
| 80%RWH-Corn | DMO17 | 2.49E+00 | 1.24E-01 | 2.01E-01 | 4.53E-04 | 4.72E-04 | 7.81E-12 | 0.05 |
| 80%RWH-Soybeans | DMO18 | 2.19E+00 | 1.09E-01 | 1.77E-01 | 3.99E-04 | 4.15E-04 | 6.87E-12 | 0.05 |
| 80%RWH-Wheat | DMO19 | 2.11E+00 | 1.06E-01 | 1.71E-01 | 3.85E-04 | 4.01E-04 | 6.64E-12 | 0.05 |
| 80%RWH-Quinoa | DMO20 | 1.22E+00 | 6.09E-02 | 9.84E-02 | 2.22E-04 | 2.31E-04 | 3.83E-12 | 0.03 |
| 100%RWH-Corn | DMO21 | 1.36E+00 | 6.92E-02 | 5.81E-04 | 2.88E-04 | 2.80E-04 | 5.82E-12 | 0.06 |
| 100%RWH-Soybeans | DMO22 | 1.20E+00 | 6.09E-02 | 5.11E-04 | 2.53E-04 | 2.46E-04 | 5.12E-12 | 0.05 |
| 100%RWH-Wheat | DMO23 | 1.16E+00 | 5.88E-02 | 4.93E-04 | 2.45E-04 | 2.38E-04 | 4.95E-12 | 0.05 |
| 100%RWH-Quinoa | DMO24 | 6.66E-01 | 3.39E-02 | 2.84E-04 | 1.41E-04 | 1.37E-04 | 2.85E-12 | 0.03 |

## **Table S7. LCIA values of well-water irrigation systems comparable to Group 2 DMOs.**

| Comparable DMO | Energy demand (MJ/m^3^) | CO_2_ emission (kg CO2 eq/m^3^) | Blue water use (m^3^/m^3^) | Ecotoxicity (CTU/m^3^) | Eutrophication (kg N eq/m^3^) | Human health- cancer (CTU/m^3^) | Life cycle costs ($/m^3^) |
| --- | --- | --- | --- | --- | --- | --- | --- |
| DMO1 | 6.99E+00 | 3.45E-01 | 1.00E+00 | 1.11E-03 | 1.24E-03 | 1.58E-11 | 0.04 |
| DMO2 | 6.15E+00 | 3.03E-01 | 8.81E-01 | 9.80E-04 | 1.09E-03 | 1.39E-11 | 0.03 |
| DMO3 | 5.94E+00 | 2.93E-01 | 8.51E-01 | 9.46E-04 | 1.05E-03 | 1.34E-11 | 0.03 |
| DMO4 | 3.42E+00 | 1.69E-01 | 4.91E-01 | 5.45E-04 | 6.08E-04 | 7.73E-12 | 0.02 |
| DMO5 | 6.99E+00 | 3.45E-01 | 1.00E+00 | 1.11E-03 | 1.24E-03 | 1.58E-11 | 0.04 |
| DMO6 | 6.15E+00 | 3.03E-01 | 8.81E-01 | 9.80E-04 | 1.09E-03 | 1.39E-11 | 0.03 |
| DMO7 | 5.94E+00 | 2.93E-01 | 8.51E-01 | 9.46E-04 | 1.05E-03 | 1.34E-11 | 0.03 |
| DMO8 | 3.42E+00 | 1.69E-01 | 4.91E-01 | 5.45E-04 | 6.08E-04 | 7.73E-12 | 0.02 |
| DMO9 | 6.99E+00 | 3.45E-01 | 1.00E+00 | 1.11E-03 | 1.24E-03 | 1.58E-11 | 0.04 |
| DMO10 | 6.15E+00 | 3.03E-01 | 8.81E-01 | 9.80E-04 | 1.09E-03 | 1.39E-11 | 0.03 |
| DMO11 | 5.94E+00 | 2.93E-01 | 8.51E-01 | 9.46E-04 | 1.05E-03 | 1.34E-11 | 0.03 |
| DMO12 | 3.42E+00 | 1.69E-01 | 4.91E-01 | 5.45E-04 | 6.08E-04 | 7.73E-12 | 0.02 |
| DMO13 | 6.99E+00 | 3.45E-01 | 1.00E+00 | 1.11E-03 | 1.24E-03 | 1.58E-11 | 0.04 |
| DMO14 | 6.15E+00 | 3.03E-01 | 8.81E-01 | 9.80E-04 | 1.09E-03 | 1.39E-11 | 0.03 |
| DMO15 | 5.94E+00 | 2.93E-01 | 8.51E-01 | 9.46E-04 | 1.05E-03 | 1.34E-11 | 0.03 |
| DMO16 | 3.42E+00 | 1.69E-01 | 4.91E-01 | 5.45E-04 | 6.08E-04 | 7.73E-12 | 0.02 |
| DMO17 | 6.99E+00 | 3.45E-01 | 1.00E+00 | 1.11E-03 | 1.24E-03 | 1.58E-11 | 0.04 |
| DMO18 | 6.15E+00 | 3.03E-01 | 8.81E-01 | 9.80E-04 | 1.09E-03 | 1.39E-11 | 0.03 |
| DMO19 | 5.94E+00 | 2.93E-01 | 8.51E-01 | 9.46E-04 | 1.05E-03 | 1.34E-11 | 0.03 |
| DMO20 | 3.42E+00 | 1.69E-01 | 4.91E-01 | 5.45E-04 | 6.08E-04 | 7.73E-12 | 0.02 |
| DMO21 | 6.99E+00 | 3.45E-01 | 1.00E+00 | 1.11E-03 | 1.24E-03 | 1.58E-11 | 0.04 |
| DMO22 | 6.15E+00 | 3.03E-01 | 8.81E-01 | 9.80E-04 | 1.09E-03 | 1.39E-11 | 0.03 |
| DMO23 | 5.94E+00 | 2.93E-01 | 8.51E-01 | 9.46E-04 | 1.05E-03 | 1.34E-11 | 0.03 |
| DMO24 | 3.42E+00 | 1.69E-01 | 4.91E-01 | 5.45E-04 | 6.08E-04 | 7.73E-12 | 0.02 |

**Descriptions of costs**

Life cycles costs of agricultural RWH system were adapted from [Ghimire and Johnston [2]](#_ENREF_2).

**Sediment chamber (pond) cost**

The cost of sediment chamber (pond) was calculated based on pond cost per unit volume, *V_p_* ($3.8/m^3^), as suggested by the Virginia FY14 Environmental Quality Incentive Program (EQIP) Payment Schedule [[3](#_ENREF_3)] (Equation S1).

*C_pond_ = 3.8* x *V_p_*  [S1]

**Tank cost**

The cost of tank (C_tank_) in $ was based on tank volume, *V_t_* (m^3^), and materials, as provided by the State of Michigan [[5](#_ENREF_5)]:

*C_tank_ =* 297.64 x *V_t_+*161.68 (if PE tank)

$=4588.9 x V_{t}^{0.5989}$ (if Concrete tank) [S2]

**Pivot center cost**

The cost of a typical pivot center, *C_pivot_* ($/ha), was derived from cost per unit of irrigated farm area, *a* [[7](#_ENREF_7)]:

*C_pivot_* = 8215.1 x *a*^-0.565^  [S3]

**Replacement cost**

Replacement occurred at the end of service life of a component: i.e., pivot center, filter, and valves were replaced at 20, 10, and 7.5 years, respectively. Present value of each replacement cost (*R_PV_)* was estimated using the corresponding single present value (SPV) discount factor:

***$R_{PV}=F_{t}\text{ x }\left(1+0.03\right)^{-t}$*** [S4]

where

*(1+0.03)^-t^* = SPV discount factor, *t* being the service life of RWH system (50 years)

0.03 = the real discount rate suggested by the National Institute of Standards and Technology [[14](#_ENREF_14)].

*F_t_* = Future price of an item such as pivot center, estimated as the sum of replacement costs at replacement times (multiples of the component’s service life less than or equal to 50). For example: for a pivot center with the base-date (2014) price of $38,296, *F_t_* was estimated as:

$F_{t}= {38,296 x \left( 1+.03 \right)}^{20}+38,296 x \left( 1+.03 \right)^{40}$ *= $194,090*

Corresponding *R_pv_* was thus estimated at $194,090 x *(1+0.03)^-50^* = $44,273.

Present value of replacement costs may also be estimated by reducing future replacement costs to the base-date at each replacement time [[2](#_ENREF_2)], which would result in a lower life-cycle cost than the method used here.

**Residual value**

Residual value of a component was estimated by the straight-line method of depreciation recommended by the Royal Institution of Chartered Surveyors and the U.S. Department of Energy [[9](#_ENREF_9), [15](#_ENREF_15)]. For example, replacement of a pivot center that operated 10 years of its 20 years of service life would have residual present value of:

 = 0.5 x $ 38,296 = $19,148

**Annual costs**

The present values of all annual costs -- operation and maintenance (O&M), sediment dredging and disposal, and pumping energy -- were estimated using uniform present value (UPV) factor. We used 1.7% of total investment costs as O&M costs for an agricultural RWH system [[13](#_ENREF_13)]. In general, sediment removal is performed once every 2-15 years, depending on pond type [[10](#_ENREF_10)], but we estimated costs of annual removal. Volume of sediment dredging was based on 0.02 m depth x 7108 m^2^ surface area per year, at $20 per cubic meter [[10-12](#_ENREF_10)]. The surface area assumed an average water depth of 1.8 m (6 ft) in a sedimentation chamber of 13000 m^3^ volume [[1](#_ENREF_1)]. Sediment disposal costs were estimated at $7/m^3^.

**Pumping Energy Costs**

Annual energy cost of pump operation was estimated utilizing the average electric rate, energy usage per cubic meter water, and volume of pumped water (Equation S5):

*C_E_ = P* x *E* x *Q*  [S5]

where

*C_E_* = Annual energy cost, $/year

*P* = Average retail price of electricity, $/kWh ($0.1030/kWh), obtained from [USEIA [16]](#_ENREF_16)

*E* = Annual energy use per cubic meter water supply for crop irrigation (0.3 kWh/m^3^), obtained from [Ghimire, Johnston [1]](#_ENREF_1)

Q = Annual pumped water (90,900 m^3^/year), obtained from [Ghimire, Johnston [1]](#_ENREF_1)

Annual pumping energy cost is a function of pumped water volume (water demand), system dynamic head, electricity price, and energy use. Water prices vary with location, block price and year, and energy price varies by fuel type, price escalation rate, and census region [[15](#_ENREF_15)], but these were not included in this analysis.

For all annual costs, we accounted for future discounting by estimating present value (PV) of annually recurring uniform amounts, as defined by:

$C_{PV}=A\text{ x }\frac{\left(1+i\right)^{n}-1}{i\left(1+i\right)^{n}}$ [S6]

where

$C_{PV}$= present value of annual cost

A = Annual costs, $

$\frac{\left(1+i\right)^{n}-1}{i\left(1+i\right)^{n}}$ *=* Uniform present value (UPV) factor

*i* = real discount rate (.03), obtained from [NIST [14]](#_ENREF_14)

*n* = number of compounding years (service life = 50 years)

# **SI2: DEA for holistic sustainability analysis**

We calculated holistic sustainability scores of the Groups 1- and 2 DMOs using modified Data Envelopment Analysis (DEA). The following steps were performed consistent with [Ghimire and Johnston [17]](#_ENREF_17):

1. Creation of a matrix of DMOs versus weights of indicators (i.e., LCIA categories) (Table S10 shows the Group 1 DMO matrix; Group 2 matrix follows the similar matrix with the corresponding number of 24 DMOs)

Initial weights were set as random values generated by Excel function as “rand()”;

The weights were denoted as: *w_1_* = Energy demand (MJ/m^3^); *w_2_* = CO_2_ emission (kg CO2 eq/m^3^); *w_3_* = Blue water use (m^3^/m^3^); *w_4_* = Ecotoxicity (CTU/m^3^); *w_5_* = Eutrophication (kg N eq/m^3^); and *w_6_* = Human health-cancer (CTU/m^3^).

1. Mean normalization of the LCIA and LCCA data (Table S11 shows the Group 1’s mean normalized data; Group 2 data follow the similar form with corresponding mean normalized data of 24 DMOs)

LCA and LCCA data were normalized using the mean-normalization method [[18](#_ENREF_18)]:

${X^{*}}_{ij}= \frac{X_{ij}}{\bar{X}_{ij}}$ [S7]

where

${X^{*}}_{ij}$= Mean normalized value of sustainability indicator *i* for *j*th DMO (dimensionless)

$X_{ij}=$ Value of sustainability indicator *i* for *j*th DMO (impact/m^3^)

$\bar{X}_{ij}=$ Average value of sustainability indicator *i* for *j* number of DMOs (impact/m^3^)

## **Table S8. Example matrix of DMO versus weights (*w_i_*) for DEA.**

| DMO | *w_1_* | *w_2_* | *w_3_* | *w_4_* | *w_5_* | *w_6_* |
| --- | --- | --- | --- | --- | --- | --- |
| DMO1 | =RAND() | =RAND() | =RAND() | =RAND() | =RAND() | =RAND() |
| DMO2 | =RAND() | =RAND() | =RAND() | =RAND() | =RAND() | =RAND() |
| DMO3 | =RAND() | =RAND() | =RAND() | =RAND() | =RAND() | =RAND() |
| DMO4 | =RAND() | =RAND() | =RAND() | =RAND() | =RAND() | =RAND() |
| DMO5 | =RAND() | =RAND() | =RAND() | =RAND() | =RAND() | =RAND() |
| DMO6 | =RAND() | =RAND() | =RAND() | =RAND() | =RAND() | =RAND() |
| DMO7 | =RAND() | =RAND() | =RAND() | =RAND() | =RAND() | =RAND() |
| DMO8 | =RAND() | =RAND() | =RAND() | =RAND() | =RAND() | =RAND() |
| DMO9 | =RAND() | =RAND() | =RAND() | =RAND() | =RAND() | =RAND() |
| DMO10 | =RAND() | =RAND() | =RAND() | =RAND() | =RAND() | =RAND() |
| DMO11 | =RAND() | =RAND() | =RAND() | =RAND() | =RAND() | =RAND() |
| DMO12 | =RAND() | =RAND() | =RAND() | =RAND() | =RAND() | =RAND() |
| DMO13 | =RAND() | =RAND() | =RAND() | =RAND() | =RAND() | =RAND() |
| DMO14 | =RAND() | =RAND() | =RAND() | =RAND() | =RAND() | =RAND() |
| DMO15 | =RAND() | =RAND() | =RAND() | =RAND() | =RAND() | =RAND() |
| DMO16 | =RAND() | =RAND() | =RAND() | =RAND() | =RAND() | =RAND() |

## **Table S9. Mean-normalized LCIA and LCCA data set.**

| DMO | Energy demand | CO_2_ emission | Blue water use | Ecotoxicity | Eutrophication | Human health-cancer | Life cycle costs |
| --- | --- | --- | --- | --- | --- | --- | --- |
| DMO1 | 2.21 | 2.23 | 1.78 | 1.75 | 2.27 | 1.89 | 1.44 |
| DMO2 | 1.84 | 1.86 | 1.49 | 1.46 | 1.89 | 1.57 | 1.20 |
| DMO3 | 1.19 | 1.21 | 0.97 | 0.95 | 1.23 | 1.02 | 0.78 |
| DMO4 | 1.06 | 1.07 | 0.85 | 0.84 | 1.09 | 0.90 | 0.69 |
| DMO5 | 2.17 | 2.22 | 1.98 | 1.71 | 2.30 | 1.90 | 1.56 |
| DMO6 | 1.81 | 1.85 | 1.65 | 1.42 | 1.92 | 1.58 | 1.30 |
| DMO7 | 1.18 | 1.20 | 1.07 | 0.93 | 1.25 | 1.03 | 0.84 |
| DMO8 | 1.04 | 1.06 | 0.95 | 0.82 | 1.10 | 0.91 | 0.75 |
| DMO9 | 0.63 | 0.59 | 0.82 | 1.09 | 0.50 | 0.90 | 1.25 |
| DMO10 | 0.52 | 0.49 | 0.68 | 0.91 | 0.42 | 0.75 | 1.04 |
| DMO11 | 0.34 | 0.32 | 0.44 | 0.59 | 0.27 | 0.49 | 0.67 |
| DMO12 | 0.30 | 0.28 | 0.39 | 0.52 | 0.24 | 0.43 | 0.60 |
| DMO13 | 0.60 | 0.57 | 1.02 | 1.05 | 0.53 | 0.91 | 1.36 |
| DMO14 | 0.50 | 0.47 | 0.85 | 0.88 | 0.44 | 0.76 | 1.13 |
| DMO15 | 0.32 | 0.31 | 0.55 | 0.57 | 0.29 | 0.50 | 0.74 |
| DMO16 | 0.29 | 0.27 | 0.49 | 0.50 | 0.25 | 0.44 | 0.65 |

1. Formulation of classical DEA

The classical DEA optimization began with standard eco-efficiency (EE) as the economic output, divided by the linear function of environmental input [[17](#_ENREF_17), [19](#_ENREF_19)]. The *n*th DMO of *N* DMOs induced *X* environmental impacts, measured by D*_nX_*. Each DMO had one economic indicator, *A_n_*.

*$Maximize\text{ }E_{n}=\frac{A_{n}}{w_{1}D_{n1}+w_{2}D_{n2}+....+w_{X}D_{nX}}\text{ (for all n = 1 to N)}$* [S8]

subject to

$\frac{A_{1}}{w_{1}D_{11}+w_{2}D_{12}+....+w_{X}D_{1X}}\leq1$ [S9]

*$\frac{A_{2}}{w_{1}D_{21}+w_{2}D_{22}+....+w_{X}D_{2X}}\leq1$* [S10]

.

.

*$\frac{A_{N}}{w_{1}D_{N1}+w_{2}D_{N2}+....+w_{X}D_{NX}}\leq1$* [S11]

*w_1_, w_2_,… w_X_$\geq$ 0* [S12]

where

*E* = holistic sustainability score

*A* = economic indicator

*D* = environmental indicator

*w_i_* = model weight estimated by DEA optimization, *i* ranges from 1 to *X*, the number of environmental and social impacts (in this example, *X* = 6)

A random number between 0 to 1 was generated as an initial value of each *w_i_*, which was then optimized by DEA. Equation S8 and the restrictions (Equations S9-S12) were non-linear functions and therefore transformed to linear form by determining the inverse functions (Equation S13-S17):

*$\text{ Minimize }E^{-1}_{n}=\frac{w_{1}D_{n1}}{A_{n}}+\frac{w_{2}D_{n2}}{A_{n}}+.....+\frac{w_{X}D_{nX}}{A_{n}}$* [S13]

subject to

*$\frac{w_{1}D_{11}}{A_{1}}+\frac{w_{2}D_{12}}{A_{1}}+.....+\frac{w_{X}D_{1X}}{A_{1}}\geq1$* [S14]

*$\frac{w_{1}D_{21}}{A_{2}}+\frac{w_{2}D_{22}}{A_{2}}+.....+\frac{w_{X}D_{2X}}{A_{2}}\geq1$* [S15]

.

.

*$\frac{w_{1}D_{n1}}{A_{n}}+\frac{w_{2}D_{n2}}{A_{n}}+.....+\frac{w_{X}D_{nX}}{A_{n}}\geq1$*  [S16]

*w_1_, w_2_,… w_X_$\geq$ 0* [S17]

The classical DEA was further modified by imposing an equal weights weighting scheme, consistent with [Ghimire and Johnston [17]](#_ENREF_17) who evaluated 10 schemes including classical DEA, equal weights, Eco-Indicator 99 [[20](#_ENREF_20)], Sustainable Society Index scheme [[21](#_ENREF_21)], National Institute of Standards and Technology (NIST) stakeholder panel scheme [[22](#_ENREF_22)], and five derived threshold schemes based on impact thresholds. They found that equal weights and thresholds overcame limitations of classical DEA by producing non-zero weights and unique sustainability scores, solving the EE non-uniqueness problem. We used equal weights, assuming each LCIA impact received equal importance.

The improved DEA formulation incorporating the equal weighting scheme was solved for each DMO, and sustainability scores were estimated. The DEA formulation of Group 1’s DMO 1 is provided as an example using corresponding mean-normalized values from Table S11 associated with each of the weights, *w_i,_* in Table S10; all other DMOs follow the similar form with corresponding *w_i_* and mean-normalized value:

For DMO 1, minimize:

$E_{DMO1}^{-1}=\frac{1}{1.44}\left( {2.21w}_{1} +{2.23w}_{2} + {1.78w}_{3} +1.75w_{4} +{2.27w}_{5} +{1.89w}_{6} \right)$ [S18]

subject to:

$\frac{1}{1.44}\left( {2.21w}_{1} +{2.23w}_{2} + {1.78w}_{3} +1.75w_{4} +{2.27w}_{5} +{1.89w}_{6} \right)\geq1$ [S19]

$\frac{1}{1.44}\left( {1.84w}_{1} +{1.86w}_{2} + {1.49w}_{3} +1.46w_{4} +{1.89w}_{5} +{1.57w}_{6} \right)\geq1$ [S20]

$\frac{1}{1.44}\left( {1.19w}_{1} +{1.21w}_{2} + {0.97w}_{3} +0.95w_{4} +{1.23w}_{5} +{1.02w}_{6} \right)\geq1$ [S21]

.

.

.

$\frac{1}{1.44}\left( {0.29w}_{1} +{0.27w}_{2} + {0.49w}_{3} +0.50w_{4} +{0.25w}_{5} +{0.44w}_{6} \right)\geq1$ [S22]

*w_1_, w_2_,… w_X_$\geq$ 0* [S23]

An improved DEA included equal weights to all impact categories (Equation S24) in addition to the classical DEA constraints, as represented in Equations S19-S23.

Equal weights (*w_X_*) for all impact categories was employed as:

*w_1_ = w_2 =_….. = w_X_* [S24]

In our case, *X* =6 and weights were: *w_1_* = Energy demand (MJ/m^3^); *w_2_* = CO_2_ emission (kg CO2 eq/m^3^); *w_3_* = Blue water Use (m^3^/m^3^); *w_4_* = Ecotoxicity (CTU/ m^3^); *w_5_* = Eutrophication (kg N eq/m^3^); *w_6_* = Human health-cancer (CTU/m^3^).

# **SI3: Basin-wide RWH sustainability indicators**

## Table S10. Net LCIA benefits of combined systems in Group 2.

| Description of DMOs | Energy demand (MJ) | CO_2_ emission (kg CO2 eq) | Blue water Use (m3) | Ecotoxicity (CTU) | Eutrophication (kg N eq) | Human health-cancer (CTU) |
| --- | --- | --- | --- | --- | --- | --- |
| 20%RWH-Corn | 7.25E+09 | 3.55E+08 | 1.29E+09 | 1.06E+06 | 1.24E+06 | 1.28E-02 |
| 20%RWH-Soybeans | 1.29E+10 | 6.32E+08 | 2.29E+09 | 1.89E+06 | 2.20E+06 | 2.28E-02 |
| 20%RWH-Wheat | 4.85E+08 | 2.37E+07 | 8.62E+07 | 7.11E+04 | 8.28E+04 | 8.57E-04 |
| 20%RWH-Quinoa | 1.61E+08 | 7.90E+06 | 2.87E+07 | 2.37E+04 | 2.76E+04 | 2.85E-04 |
| 40%RWH-Corn | 1.45E+10 | 7.10E+08 | 2.58E+09 | 2.13E+06 | 2.48E+06 | 2.56E-02 |
| 40%RWH-Soybeans | 2.58E+10 | 1.26E+09 | 4.59E+09 | 3.78E+06 | 4.41E+06 | 4.56E-02 |
| 40%RWH-Wheat | 9.69E+08 | 4.75E+07 | 1.72E+08 | 1.42E+05 | 1.66E+05 | 1.71E-03 |
| 40%RWH-Quinoa | 3.23E+08 | 1.58E+07 | 5.74E+07 | 4.73E+04 | 5.51E+04 | 5.71E-04 |
| 60%RWH-Corn | 2.17E+10 | 1.06E+09 | 3.87E+09 | 3.19E+06 | 3.71E+06 | 3.85E-02 |
| 60%RWH-Soybeans | 3.87E+10 | 1.89E+09 | 6.88E+09 | 5.67E+06 | 6.61E+06 | 6.84E-02 |
| 60%RWH-Wheat | 1.45E+09 | 7.12E+07 | 2.59E+08 | 2.13E+05 | 2.48E+05 | 2.57E-03 |
| 60%RWH-Quinoa | 4.84E+08 | 2.37E+07 | 8.61E+07 | 7.10E+04 | 8.27E+04 | 8.56E-04 |
| 80%RWH-Corn | 2.90E+10 | 1.42E+09 | 5.16E+09 | 4.25E+06 | 4.95E+06 | 5.13E-02 |
| 80%RWH-Soybeans | 5.16E+10 | 2.53E+09 | 9.17E+09 | 7.56E+06 | 8.81E+06 | 9.12E-02 |
| 80%RWH-Wheat | 1.94E+09 | 9.50E+07 | 3.45E+08 | 2.84E+05 | 3.31E+05 | 3.43E-03 |
| 80%RWH-Quinoa | 6.45E+08 | 3.16E+07 | 1.15E+08 | 9.46E+04 | 1.10E+05 | 1.14E-03 |
| 100%RWH-Corn | 3.62E+10 | 1.77E+09 | 6.45E+09 | 5.31E+06 | 6.19E+06 | 6.41E-02 |
| 100%RWH-Soybeans | 6.45E+10 | 3.16E+09 | 1.15E+10 | 9.46E+06 | 1.10E+07 | 1.14E-01 |
| 100%RWH-Wheat | 2.42E+09 | 1.19E+08 | 4.31E+08 | 3.55E+05 | 4.14E+05 | 4.29E-03 |
| 100%RWH-Quinoa | 8.06E+08 | 3.95E+07 | 1.43E+08 | 1.18E+05 | 1.38E+05 | 1.43E-03 |

## **Fig S1. Sensitivity of sustainability indicators of combined systems.**

In Fig S1, the percentage (%) benefits were estimated with respect to 60% RWH-Soybeans system. The variation in basin-wide sustainability indicators (or life cycle benefits) due to the combined agricultural systems (combined RWH and well-water) relative to well-water irrigation ranged from 33% to 167% (Fig S1). RWH-Soybeans system is shown as an example but similar relationship was observed for all combined RWH-crop system as well.

## **Fig S2. Sensitivity of sustainability indicators of RWH-Quinoa system to quinoa crop area.**

In Fig S2, the percentages (%) were computed with respect to RWH-Quinoa system with originally assumed quinoa area at 226,698,300 m^2^. Sensitivity analysis of the basin-wide sustainability indicators of RWH-Quinoa agricultural system showed linear relationships (Fig S2).

## **Fig S3.** **Sensitivity of sustainability indicators of combined systems to system service life.**

In Fig S3, the percentages (%) were computed with respect to a hypothetical combined system of 0.4 well-water:0.6RWH with the original system service life at 50 y. Sensitivity analysis of the basin-wide sustainability indicators to service life showed linear relationships. Basin-wide sustainability indicator of life cycle energy demand savings are shown as an example but similar trends were observed for other indicators as well.

Sensitivity of sustainability indicators to adoption rates also showed a linear relationship, similar to the service life.

# **REFERENCES**

1. Ghimire SR, Johnston JM, Ingwersen WW, Hawkins TR. Life Cycle Assessment of Domestic and Agricultural Rainwater Harvesting Systems. Environmental Science & Technology. 2014;48(7):4069-77. doi: 10.1021/es500189f.

2. Ghimire SR, Johnston JM. Holistic impact assessment and cost savings of rainwater harvesting at the watershed scale. Elem Sci Anth. 2017;5.

3. USDA. FY14 EQIP Payment Schedule. USA: United States Department of Agriculture 2014.

4. CPFC. Price Lists USA: Charlotte Pipe and Foundry; 2014 [March 26, 2014]. Available from: <http://www.charlottepipe.com/price_lists.aspx>.

5. SOM. Tanks Section UIP 11. USA: State of Michigan, 2003.

6. Pentair. BERKELEY® centrifugal pumps and accessories U.S. 2014 list prices USA: Pentair Ltd.; 2014 [March 28, 2014]. Available from: <http://www.berkeleypumps.com/ResidentialResourceCenter.aspx>.

7. Bliesner RD, Spare D, editors. Center Pivot Irrigation. Second Annual Four Corners Irrigation Workshop 2001; USA: New Mexico State University.

8. Flomatic. Flomatic® Valves Price List March 24, 2014 USA: Flomatic Corporation; 2014 [March 27, 2014]. Available from: <http://www.flomatic.com/>.

9. RICS. RICS draft guidance note: Life cycle costing The United Kingdom: Royal Institution of Chartered Surveyors 2014 [cited 2014 Feb. 21, 2014]. Available from: <https://consultations.rics.org/consult.ti/life_cycle_costing/viewCompoundDoc?docid=794580&sessionid=&voteid=&partId=795956>.

10. Commission NVR. Maintaining Stormwater Systems: A Guidebook for Private Owners and Operators in Northern Virginia. Fairfax: Virginia Coastal Zone Management Program at the Department of Environmental Quality; 2007.

11. Marsalek J, Marsalek P. Characteristics of sediments from a stormwater management pond. Water Science and Technology. 1997;36(8):117-22.

12. USDA. Ponds — Planning, Design, Construction. USA: Natural Resources Conservation Service (NRCS), 1997.

13. Hogan R, Stiles S, Tacker P, Vories E, Bryant K. Estimating irrigation costs: Cooperative Extension Service, University of Arkansas, US Department of Agriculture, and county governments cooperating; 2007.

14. NIST. Energy Price Indices and Discount Factors for Life-Cycle Cost Analysis – 2013 Annual Supplement to NIST Handbook 135 and NBS Special Publication 709. National Institute of Standards and Technology, U.S. Department of Commerce; 2013.

15. Fuller S, Petersen S. Life-cycle costing manual for the federal energy management program, 1995 Edition. NIST handbook. 1996;135.

16. USEIA. Electricity USA: U.S. Energy Information Administration (EIA); 2014 [Jan. 22, 214]. Available from: <http://www.eia.gov/electricity/data.cfm#sales>.

17. Ghimire SR, Johnston JM. A modified eco‐efficiency framework and methodology for advancing the state of practice of sustainability analysis as applied to green infrastructure. Integrated Environmental Assessment and Management. 2017.

18. Sarkis J. Preparing your data for DEA. In: J. Z, W. C, editors. Modeling data irregularities and structural complexities in Data Envelopment Analysis. New York, NY: Springer; 2007. p. 305-20.

19. Kuosmanen T, Kortelainen M. Measuring Eco‐efficiency of Production with Data Envelopment Analysis. Journal of Industrial Ecology. 2005;9(4):59-72.

20. Goedkoop M, Spriensma R. The Eco-indicator 99: a damage-oriented method for life cycle impact assessment, methodology report. PRé Consultants, Amersfoort, The Netherlands, 2000.

21. Sironen S, Seppälä J, Leskinen P. Towards more non-compensatory sustainable society index. Environment, Development and Sustainability. 2014:1-35.

22. Gloria TP, Lippiatt BC, Cooper J. Life cycle impact assessment weights to support environmentally preferable purchasing in the united states. Environmental Science & Technology. 2007;41(21):7551-7. doi: 10.1021/esO7O750+. PubMed PMID: WOS:000250556100059.
